# Decarbonizing real estate portfolios considering optimal retrofit investment and policy conditions to 2050

**DOI:** 10.1016/j.isci.2023.106619

**Published:** 2023-04-08

**Authors:** Ivalin Petkov, Alicia Lerbinger, Georgios Mavromatidis, Christof Knoeri, Volker H. Hoffmann

**Affiliations:** 1 Group for Sustainability and Technology, ETH Zurich, 8092 Zurich, Switzerland; 2 OPTIML AG, 8003 Zurich, Switzerland

**Keywords:** Energy resources, Energy policy, Energy Modeling, Energy flexibility

## Abstract

Retrofitting existing buildings is crucial for achieving Net Zero emissions. Institutional real estate owners play a key role because of their significant ownership, especially of large buildings. We utilize an interdisciplinary approach to evaluate cost-optimal decarbonization conditions for three Swiss real estate portfolios owned by a global institutional investor. We leverage a bottom-up optimization framework for building asset retrofitting, scaled to the portfolio-level, to study the effect of policy scenarios and implementations. Results indicate that achieving Net Zero necessitates significant investments, largely through thermal energy efficiency measures and low-CO_2_ energy systems, as early as possible to avoid locked-in emissions. Owners will be challenged to smooth long-term capital investments, pointing to a potential liquidity crisis. Consequently, hard-to-decarbonize assets are unable to reach regulatory benchmarks largely because of lingering embodied emissions. To lower transition risk, we recommend that policymakers move toward average CO_2_ benchmarks at the real estate portfolio-level, emulating automotive fleets.

## Highlights

• Interdisciplinary approach to evaluate cost-optimal real estate decarbonization
• Bottom-up existing building retrofitting models scaled to the portfolio level
• Achieving whole life Net Zero requires significant capital to avoid carbon lock-in
• Policymakers play a role in innovating policy implementation to lower transition risk

## Graphical abstract

**Figure:**
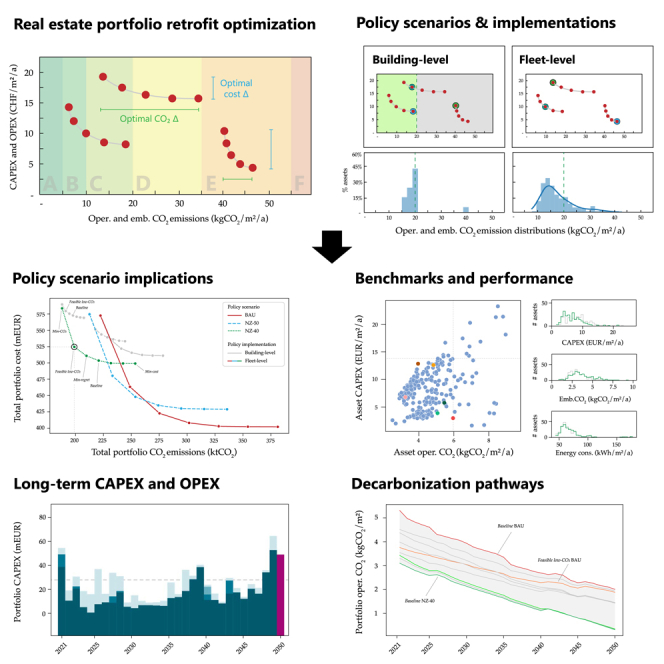

## Introduction

Decarbonizing the buildings and construction sector, responsible for 36% of energy consumption and 39% of energy-related CO_2_ emissions globally in 2018,^1^ is crucial to achieve the 1.5°C climate goal. Various science-based targets for buildings exist which are in line with global IPCC pathways.^2^^,^^3^ One such target by the World Green Building Council (WGBC)^4^ encompasses buildings’ whole-life operational and embodied carbon footprint and aims for Net Zero by 2050. Continued urban growth, largely in developing economies, is expected to double global floor area by 2050,^5^ presenting difficulties to achieve the target.

Two challenges exist concerning decarbonization: (1) Assuring that *new buildings* are efficient, resilient, energetically renewable, while being constructed with low-CO_2_ footprint materials, and (2) addressing the aging *existing building* stocks of developed economies, such as Europe’s, where 90% of buildings are still expected to stand in 2050.^6^^,^^7^ With new building regulations in Europe approaching Net Zero operational CO_2_ by 2030,^8^ targets are primarily threatened by low retrofitting rates (<1% annually).^9^ The rate of *deep retrofits* must increase up to 3%,^10^^,^^11^ encompassing a combination of energy efficiency (EE), renewable energy (RE), and complementary technologies (e.g. heat pumps and batteries). For buildings, these options are both commercially-available and commonplace.^12^

Institutional real estate owners play a crucial role to achieve a Net Zero building stock because of their (1) significant ownership, (2) large investment shares, (3) centralized decision-making, and (4) available capital.^13^^,^^14^^,^^15^ Owners are being pressured to incorporate Environmental, Social, and Governance (ESG) criteria, and CO_2_ performance specifically, into investment strategies.^16^^,^^17^ Voluntary reporting mechanisms^14^ currently focus only on operational CO_2_ emissions—*Scope 1* (direct emissions from combustion) and *Scope 2* (grid imports of electrical and thermal energy)—but embodied emissions of materials and technologies (*Scope 3*) is ignored.^18^ Considering CO_2_ is compelling real estate owners to develop bespoke action plans for *specific portfolios*.^4^ Owing to the uniqueness of *each building asset,* they must systematically re-evaluate retrofitting potentials, technological options, and estimate policy-relevant transition risk.^19^

Policies in the energy, climate, and real estate market domains influence real estate owners’ investment decisions in retrofits.^20^ The complex interactions of these policies, and their possible developments, challenge policymakers to set consistent and coherent policy mixes^21^ toward Net Zero. Taken together, both actors’ decisions are interdependent for meeting long-term CO_2_ targets: (1) Owners’ alignment of unique asset strategies across portfolios, and (2) policymakers’ settings of policy conditions across various instruments. In this study, we take an interdisciplinary approach to deliver both policy and managerial perspectives toward the research question: *Under which conditions can real estate portfolios be cost-optimally decarbonized?* We do so by optimizing long-term retrofitting investment strategies to evaluate the real estate portfolio Net Zero transition.

Portfolio action plans navigate the large decision-space regarding *what* retrofitting project to do, *when* to prioritize investments, on which building (*where*), and *how* that impacts economic and environmental performance in future conditions. Extant methodologies used to develop asset to portfolio retrofitting plans,^22^^,^^23^ prominently the Carbon Risk Real Estate Monitor (CRREM),^24^^,^^25^ are largely limited in temporal, technological, and spatial dimensions. Generally, they utilize benchmarks and top-down retrofitting assumptions, but do not: (1) accurately account for each assets’ context at the portfolio-level,^26^ (2) develop investment strategies to 2050 subject to trade-offs of cost and Scope 1–3 CO_2_,^27^^,^^28^^,^^29^ (3) consider the interactions of the large set of technological options on buildings’ energy demand and supply,^30^ and (4) evaluate future developments such as binding policies, economic contexts, and technological improvements.^31^^,^^32^ Without considering these aspects, the available option-space is limited, making it difficult to find an optimal solution to the complex real estate investment and decarbonization decision-making problem.

**Figure 1:**
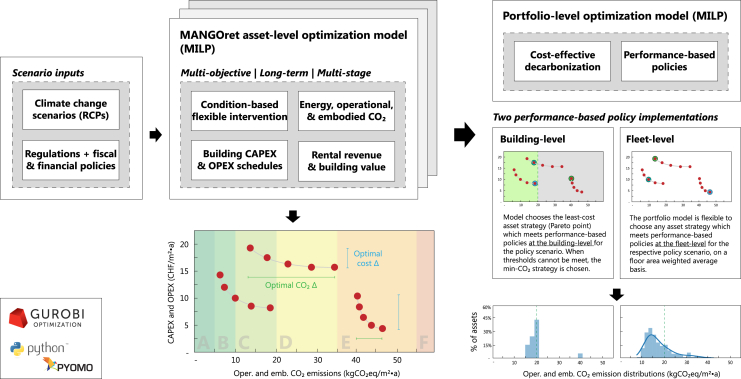
Graphical depiction of asset to portfolio model workflow Pareto fronts for the total aggregated portfolio 30-year costs and CO_2_ emission with RCP 4.5. The Paretos are varied across policy conditions: three policy scenarios under different performance-based policy implementation approaches: building-level (gray) versus fleet-level (colors).

We utilize a two-step approach: first, we use MANGOret (Multi-stAge eNerGy Optimization — retrofitting), a bottom-up building optimization model framework relevant for comprehensive retrofitting planning.^33^ The model conducts a multi-objective cost (Net Present Value) and lifecycle CO_2_ optimization for all aspects of asset retrofitting strategies: materials to technologies and systems. The asset strategies are fed into a portfolio optimization model to generate cost-optimal decarbonization plans to 2050 (see STAR Methods for details with graphical depiction in Figure 1).

We incorporate a dynamic dataset including (1) climatic Relative Concentration Pathways (RCPs), (2) techno-economic and context parameter developments, and (3) policy conditions. To better establish the energy modeling—policy interface,^34^ we evaluate policy conditions by constructing three comprehensive building sector policy mix^21^ scenarios along with further analyzing the influence of an automotive fleet-inspired policy implementation approach for building portfolios. We differ from existing international-level analyses,^35^^,^^36^ national-level analyses^37^^,^^38^^,^^39^^,^^40^^,^^41^^,^^42^ and scenario analyses,^43^^,^^44^^,^^45^^,^^46^ which largely consider cost optimization toward generalized energy or emissions benchmarks, by taking an owner’s investment perspective under various policy conditions.

Our case study, presented in Table 1, encompasses 2020 data for three real estate portfolios of global institutional investors, with all assets domiciled in Switzerland. The three portfolios are diversified across real estate markets with building types, uses, sizes, and ages, being representative of other European institutional real estate investor portfolios. Overall, we consider an aggregated portfolio of 235 assets comprising 600 buildings with an average age of 45 years, with the uses: multi-family residential homes (55%), office (25%), retail (15%), hotel (3%), and other (2%). The portfolios have a value of nearly EUR 6.25b and 1.55 million m^2^ floor area.

**Table 1:** Real estate portfolio case study basic data

Portfolio	Value (bEUR)	Assets	Bldgs.	Ave. build yr.	Floor area (m^2^)	Usage^a^				
						MFH	Office	Retail	Hotel	Other
1	3.00	150	400	1970	700,000	80%	10%	5%	2%	3%
2	1.25	50	100	1970	250,000	25%	35%	30%	5%	5%
3	2.00	35	100	2010	600,000	40%	35%	15%	5%	5%
**Agg.**	**6.25**	**235**	**600**	**1975**	**1,550,000**	**55%**	**25%**	**15%**	**3%**	**2%**

a Usage is reported in weighted floor area from Global Real Estate Sustainability Benchmark (GRESB) categories. Other building usage comprises Manufacturing, Lodging & Leisure, Medical Office, and Restaurant & Bar. Map of asset locations are shown in Data S1. To assure anonymity, values are rounded. Portfolio data histograms are presented in Figure S5.

Our results indicate that future-looking policy scenarios present significant cost and CO_2_ emission trade-offs for real estate portfolio decarbonization. Moving toward fleet-level CO_2_ benchmarks away from the current Building Energy Code “one-size-fits-all” approach, similar to the average requirements for automotive fleets, could help to cost-effectively reach low-CO_2_ for real estate portfolios and reduce stranded asset risks.

We find that deep retrofits are urgently necessary to decarbonize, requiring increased investments from owners into envelope thermal EE investments and RE-based heating systems. The increased capital expenditure required to avoid a “carbon bubble”, defined as real estate assets not strategized to meet decarbonization goals consequently locking-in emissions,^47^ points to a potential liquidity crisis in the industry. The carbon impacts largely lie with embodied emissions of retrofits, for which there are currently few technological alternatives, thus making achieving Net Zero heavily reliant on offsetting as a last resort.

### Policy conditions description

We develop two sets of policy conditions: (1) scenarios and (2) implementation approaches. The three policy scenarios consider the long-term evolutions of over ten different policy instruments relevant for European buildings from the energy, climate, and real estate domains with national energy scenarios. As different policy regimes can influence investment strategies, we used the prominent rationale used in scenario development methods^48^^,^^49^ to partner the current goal of *Net Zero 2050* (NZ-50) with two extreme scenarios—*Business-as-usual* (BAU) and *Net Zero 2040* (NZ-40). The policy scenarios and instruments are presented in Table 2.

**Table 2:** Specific policy instrument settings for the three policy mix scenarios

		Business-*as*-usual	Net Zero 2050	Net Zero 2040
		BAU	NZ-50	NZ-40
Regulations				
Building energy code	Thermal energy efficiency req. in relation to new buildings (kWh/m^2^) ∗	**36** [24–38]	**30** [20–31]	**24** [16–25]
	CO_2_ performance req. (kgCO_2_/m^2^) ∗	–	20	10
	On-site electricity production req. (W/m^2^) ∗	–	5	10
	Renewable heat req. (%) ∗	10%	40%	80%
	Fossil heating tech. bans (oil & gas boilers)	–	oil	oil & gas
Real estate	Portfolio reinvestment req. (%)	2%	4%	6%
	Component pass-on rates (%)	2020 levels	−10%	+10%
Financial incentives and fiscal instruments				
CO_2_tax	CO_2_ tax on fossil fuels (EUR/tonCO_2_)	Constant at 120 (2020 level)	Linear increase from 120 to 168	Linear decrease from 145 to 97
Incentives	Incentives per retrofitting component, renewable heating, solar PV, and batteries (fixed & linear)	2020 levels	+50%	+100%
Swiss Energy Strategy 2050 context parameters				
Energy prices	Energy carrier prices (EUR/kWh)			
	Feed-in-tariff for solar PV export to grid (EUR/kWh)	Political Measures (POM) scenario with grid CO_2_ factor differentiated		New Energy Policy (NEP) scenario
Grid CO_2_ factor	Electricity grid CO_2_ factor (kgCO_2_/kWh)			

More detailed descriptions of the policy mix narratives, development, and instrument evolutions from 2021 to 2050 are provided in the Supplementary Information. All policies are implemented as constants unless otherwise noted. Performance-based instruments (∗) are implemented in the portfolio-level optimization model to conduct the fleet-versus building-level policy implementation approaches.

The implementation approaches pertain to how policies toward decarbonization are enforced in the building fleet. Next to the presently used regulation on a building-level in all Building Energy Code (BEC) regimes globally and in Europe,^8^ we take inspiration from automotive fleet-level CO_2_ regulations to differentiate two policy implementation approaches for performance-based policies—thermal energy efficiency, CO_2_ performance, renewable heating, and on-site electricity production requirements. For example, automobile manufacturers are regulated at both (1) standard for individual vehicle types (e.g. cars, vans, and trucks) and (2) fleet-wide average requirements such as Europe’s 2020 goal of 95 gCO_2_/km.^50^^,^^51^^,^^52^

We use this as an example for the real estate context: this would mean enforcing performance metrics for (1) each building in an asset versus (2) a weighted average value for the entire portfolio. In this article, the building-level policy approach is implemented at the level of a real estate asset which is often just one building but could also comprise several similar buildings next to each other. The fleet-level policy approach is implemented at the level of the real estate portfolio, which is an aggregation of assets. This approach could be expanded to a larger scope to consider a fleet of buildings in a city, region, and country. Taken together, we first optimize retrofitting strategies for each asset on a multi-objective basis from minimum-cost to minimum-CO_2_ considering the three policy scenarios. Next, we optimize portfolio plans based on the optimal asset strategies considering the two policy implementation approaches.

## Results

### Policy influence on optimal decarbonization strategies

**Figure 2:**
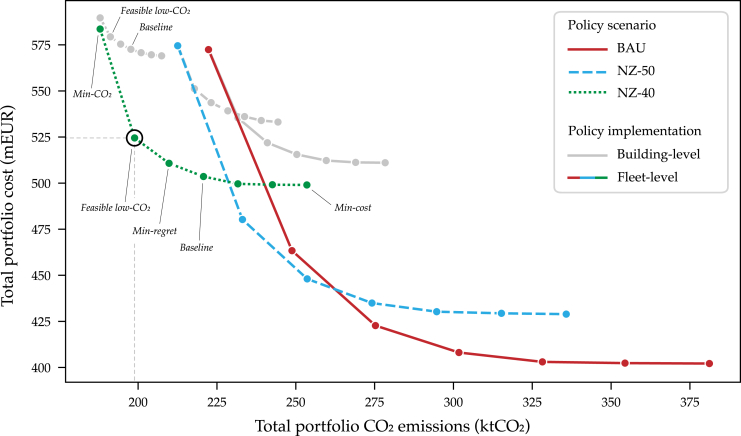
Optimal portfolio strategies under policy conditions (A) Comparison of asset-level results between policy implementation approaches in the NZ-40 scenario for the *Baseline* (building-level) and the *Feasible low-CO*_*2*_ (fleet-level) strategies. Asset’s average annual CAPEX investment and CO_2_ emissions are shown. Six assets are highlighted in color to illustrate their movement between the strategies, categorized as: “hard-to-decarbonize” moving to higher CO_2_ with cost decrease (down and right): brown and yellow, “low-hanging-fruit” moving to lower CO_2_ with little cost increase (left): dark and light green, “no change”: red and pink. (B) For the same two strategies, the difference of buildings’ contributions to portfolio total CAPEX and CO_2_ emissions are shown. Here, more negative values flag expensive hard-to-decarbonize buildings while less negative values are “low-hanging-fruit” from a cost and CO_2_ standpoint. (C) Histogram distributions of average annual CAPEX, CO_2_ emissions scopes, and energy consumption between strategies (over the 30-year horizon) including both building- (gray) and fleet-level results (green).

The influence of policy conditions on the cost to CO_2_ optimal portfolio strategies is shown in Figure 2. The option ranges, termed Pareto fronts, have strategic points which represent 30-year performance. Owing to the attractive cost-effective emissions reductions from *Min-cost* toward the left in the Pareto, in the following we term the fourth strategy (middle) as the *Baseline*. As reducing emissions becomes more expensive, for ease of recognition we colloquially term the fifth point as the *Min-regret* strategy, the sixth point as the *Feasible low-CO*_*2*_ strategy, followed by the *Min-CO*_*2*_ strategy. We find that climatic RCP scenarios have little impact on portfolio costs and emissions, generally differentiating costs +/− 4% within each policy scenario Pareto. Here we present results only for RCP 4.5, with RCP 8.5 insights presented in Figure S1.

Comparing the extreme strategies (*Min-cost* BAU versus *Min-CO*_*2*_ NZ-40, both fleet-level), total costs without considering incentives increase 45% (with incentives, 25%) with subsequent lifecycle emissions reductions of 47%. In other words, the modeled scenario futures present a large option-space with cost and CO_2_ implications for owners’ investment strategies. To commit to an optimal strategy, an owner has to “believe” that this future would be possible, especially a low-CO_2_ one that entails high capital expenditure (CAPEX) on efficient and renewable retrofits. This affirms importance of reliability and clarity of future policy developments to aid investment decision-making.

Toward more stringent policy scenarios, the Pareto fronts get tighter both from a cost and CO_2_ perspective. However, the option-space is far more reduced by the policy implementation approaches than the policy scenarios. Within each scenario, the building-level policy implementation Paretos (gray) are always sub-optimal in relationship to the fleet-level (colors): For similar levels of emissions in the *Feasible low-CO*_*2*_ NZ-40 strategy, the fleet-level approach reduces costs by EUR 48m (8%). Although, because of the higher flexibility, the fleet-level also presents higher emitting yet lower-cost solutions. As with other energy assets,^53^^,^^54^ a fleet-level approach presents more attractive solutions for achieving low-CO_2_ cost-effectively. Such an approach shows promise for innovating beyond performance requirements regulated solely at the building-level.

### Beyond benchmarks toward fleet-level approaches

**Figure 3:**
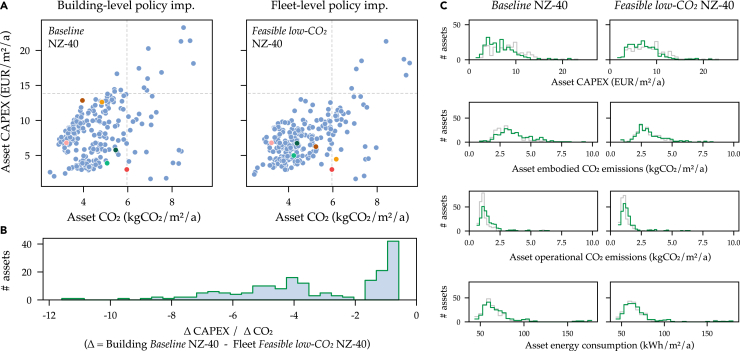
Asset-level performance across policy implementation approaches Individual assets’ optimal cost and CO_2_ results over 30-year are presented as steps. Comparison between the *Baseline* (solid) and *Feasible low-CO*_*2*_ (dotted) portfolio strategies for the BAU (red) and NZ-40 (green) policy scenarios (RCP 4.5, fleet-level policy implementation).

As real estate portfolios comprise diverse assets, Figure 3 presents a granular view of key asset cost, CO_2_, and energy consumption metrics for the NZ-40 scenario *Baseline* (building-level) and *Feasible low-CO*_*2*_ (fleet-level). We compare these strategies as they have the same CO_2_ emissions, but the *Baseline* (building-level) strategy has 9% higher cost. Results for *Baseline* (fleet-level) and *Feasible low-CO*_*2*_ strategies (building-level) are presented in Figures S2 and S3.

Unlike automobiles, each building asset is unique because of its distinctive starting conditions and existing systems, presenting different energy and CO_2_ reduction potentials. The fleet-level policy approach grants a certain decision-making flexibility at the asset-level, as shown in the top-right quadrants of Figure 3A where there are fewer “expensive and CO_2_-heavy” assets. This way, the fleet-level approach optimally prioritizes which assets are strategically “hard-to-decarbonize”^55^ and “low-hanging-fruit”. The relative movement by the low-hanging-fruits to decrease CO_2_ outweighs the cost increases from the hard-to-decarbonize buildings, overall leading to a lower-cost portfolio strategy for the same emissions. Figure 3B visualizes the definitions of assets’ movement between the strategies: The hard-to-decarbonize assets which increase in CO_2_ but reduce CAPEX (values smaller than −2) and low-hanging-fruit which decrease in CO_2_ but increase CAPEX (peak between −1.7 and 0).

In other words, regulating at defined benchmarks for CO_2_ and energy consumption with a “one-size-fits-all” for *all building assets* pushes owners toward higher investment in low-CO_2_ building technologies, even with similar total CO_2_. Policymakers could play a role in cost-effective decarbonization by allowing owners more investment flexibility to leverage portfolio decisions considering each asset’s optimal option space. Otherwise, owners face a higher risk of stranded assets.

In the NZ-40 scenario, with the stringent combination of thermal EE (<24 kWh/m^2^) and renewable heating (>80%), some assets could be labeled as stranded on a CAPEX-basis (>14 EUR/m^2^/a) or on a CO_2_-basis (>6 kgCO_2_/m^2^/a). Stranding refers to assets which are outliers in a portfolio strategy under a certain policy scenario, considered as two standard deviations from the mean. Owners might consider selling stranded assets to a non-GRESB reporting owner—brown-spinning^56^—or otherwise choose to demolish and rebuild, leading to significantly higher emissions than retrofitting.^18^ Such situations present the danger of overall increasing building sector emissions and could be prevented through the fleet-level approach. This could be especially beneficial for valuable assets for which there are few cost-effective options to reduce energy and CO_2_ because of the contextual situation: building use, construction quality, or historical protection.

Investment flexibility at fleet-level is demonstrated in the Figure 3C distributions of both building-level (gray) and fleet-level (color) asset results for the NZ-40 *Baseline* and *Feasible low-CO*_*2*_ strategies. Here, cost-benefits are largely achieved by lower CAPEX shown by median movements of -22% (*Baseline*) and -15% (*Feasible low-CO*_*2*_), however with a wide distribution between assets. Taking the example of *Feasible low-CO*_*2*_ NZ-40, the decreased CAPEX at the median is counter-balanced by much smaller median increases in OPEX (1%), energy (2%), with both operational (14%) and embodied (4%) emissions. However, it must be noted that total emissions remain similar between fleet- and building-level policy implementations.

The asset distributions of embodied emissions and energy consumption are much wider than operational emissions for two reasons: (1) The limited decision space between envelope retrofitting technologies which dictate thermal energy demands, and (2) the techno-economic attractiveness to reduce operational CO_2_ by adapting energy systems’ design and operation.

**Figure 4:**
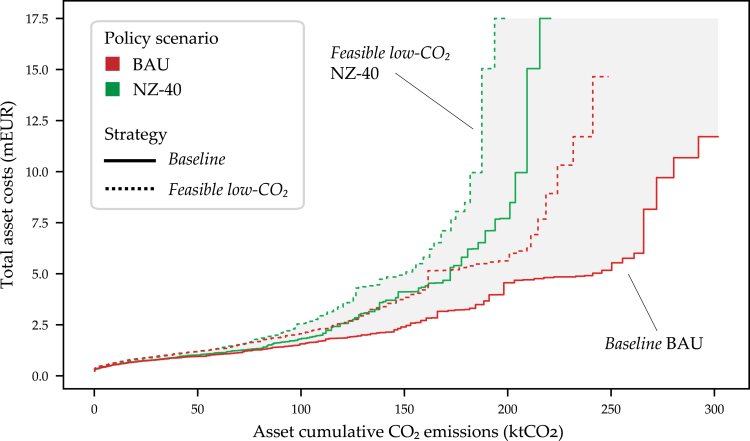
Merit order of cost-optimal decarbonization across policy scenarios (A) Total 30-year discounted cost and CO_2_ emissions scope contributions across strategies and policy scenarios (RCP 4.5, fleet-level policy implementation). Revenues from incentives, feed-in-tariff, and salvage presented as negative costs. (B) Long-term nominal CAPEX investments per technology category for two *Feasible low-CO*_*2*_ strategies between the BAU and NZ-40 policy scenarios (labeled with ∗).

Another perspective of strategic flexibility for real estate portfolio decarbonization is represented in the Figure 4 merit order. Moving toward more stringent low-CO_2_ strategies shows divergence between chosen asset strategies, with the many assets in the left to middle of the curves gradually increasing in costs to achieve lower emissions. The pronounced difference lies on the right side of the merit order, with a fewer number of assets contributing a disproportionate amount of cost.

Five large assets (160,000 m^2^ floor area, 10% of portfolio) contribute to around 13% for both cumulative CO_2_ emissions and costs in the *Baseline* BAU and *Feasible low-CO*_*2*_ NZ-40 strategies. However, the absolute values for the strategic differences for these five assets differ significantly by 16 ktCO_2_ and EUR 13m: *Baseline* BAU (40 ktCO_2_, EUR 46m) versus *Feasible low-CO*_*2*_ NZ-40 (24 ktCO_2_, EUR 59m). Although these five assets present large contributions to total portfolio cost and CO_2_ budgets, nevertheless on a per floor area basis, they are considered CAPEX low-hanging-fruits with very high operational emissions and energy demands. Generalized, the portfolio approach tends to increase investments in large assets to reduce their CO_2_ footprints while letting smaller buildings increase their emissions. This gives important insights for carbon transition risk with certain assets contributing differently toward portfolio decarbonization.

### Urgent deep retrofits reduce carbon bubbles

For owners, planning for long-term decarbonization necessitates scheduling CAPEX investments for a large set of interdependent building technologies and systems: EE measures, RE, conversion, and storage technologies with non-energy renovations. Our results show that it is imperative that immediate action is taken to reduce energy-use and CO_2_-intensity of existing buildings to achieve low-CO_2_ cost-optimally.

**Figure 5:**
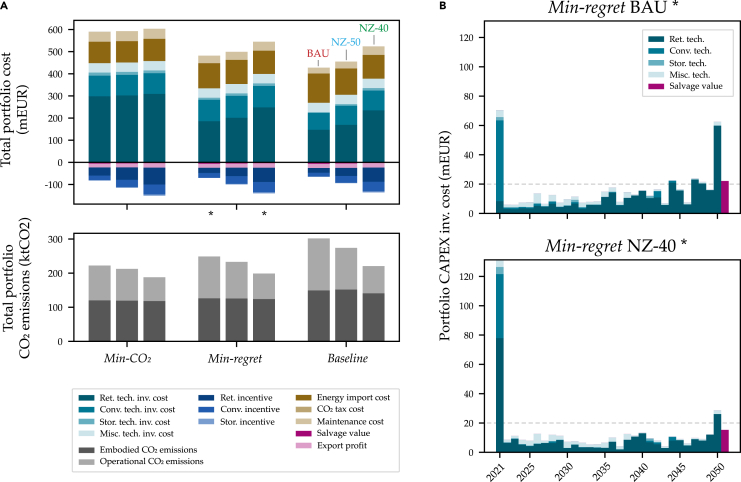
Portfolio investment cost compositions and schedules with CO_2_ emissions scopes Life cycle operational CO_2_ emission pathways (Scope 1 & 2) for the aggregated portfolios compared between the *Baseline* BAU (red), *Feasible low-CO*_*2*_ BAU (orange), *Baseline* NZ-40 (light green), and *Feasible low-CO*_*2*_ NZ-40 (dark green) scenarios (RCP 4.5, fleet-level policy implementation). All other scenarios lie in the gray zone between. Total contributions of all energy carriers toward 30-year operational CO_2_ emissions shown on right.

Figure 5 shows the costs incurred in optimal strategies across the various policy scenarios and under the fleet-level policy implementation approach. Higher costs are largely because of early investments in envelope retrofits (for owners, the largest CAPEX category with over 34% of total costs in *Min-regret* strategies) and low-CO_2_ energy systems (17% of total costs). Optimal low-CO_2_ strategies take advantage of the opportunity to achieve more energy efficient buildings right away owing to possible thermal energy reductions of up to 50–80% from deep retrofits (Figure S6). In addition, the strategies benefit from adequately-sized RE-based systems and thus avoid a “carbon bubble” by re-strategizing away from inefficient and fossil-reliant buildings.

In comparison, OPEX retains a relatively constant share of total costs (38%) across strategies which are usually paid by tenants. Incentives for retrofitting and conversion technologies in the NZ-40 and NZ-50 scenarios buffer some of owners’ net CAPEX investments. Although net CAPEX is still higher between the BAU and NZ-40 for *Baseline* (15%) and *Min-regret* (9%), it is lower by 16% in the *Min-CO*_*2*_.

Although the presented low-CO_2_ optimal strategies could reduce the size of the carbon bubble, they could still position the real estate sector at the risk of a liquidity crisis. Owners will be challenged to smooth portfolio CAPEX investments over the years to avoid large “spikes” with many buildings necessitating retrofits at once. Comparing the *Min-regret* BAU and NZ-40 strategies in Figure 5B, the optimal solution demonstrates a nearly double 2021 CAPEX spike.

To reach Net Zero, the large impact of embodied emissions will need to be managed as they account for the majority in low-CO_2_ strategies (50–60%). Insulation materials and window components, with energy technologies such as solar PV, are the main culprits and are difficult to avoid because of technologically immature alternatives^57^ and lacking circular economy systems.^58^ The 2021 CAPEX spike also yields an embodied “carbon spike”^59^ in low-CO_2_ strategies. This is largely because of attractive solar PV installations in the *Min-regret* BAU strategy with deep retrofits in the *Min-regret* NZ-40 strategy.

Although our results demonstrate the techno-economic feasibility of decarbonization for all scopes on a life cycle basis, nevertheless significant emissions remain. Reliable offsetting measures will have to be taken to achieve Net Zero. Illustratively, offsetting the remaining 209 ktCO_2_ in the *Min-regret* NZ-40 strategy with Direct Air Capture would increase owners’ costs by EUR 63m (16%) assuming an average future cost of 300 EUR/ton CO_2_ abated.^60^

Although embodied emissions have the highest shares, the long-term decarbonization of a portfolio relies on operational emission reductions. In the following, we break down the contributing elements for both operational and embodied emissions.

### Operational CO_2_ decarbonization pathways

**Figure 6:**
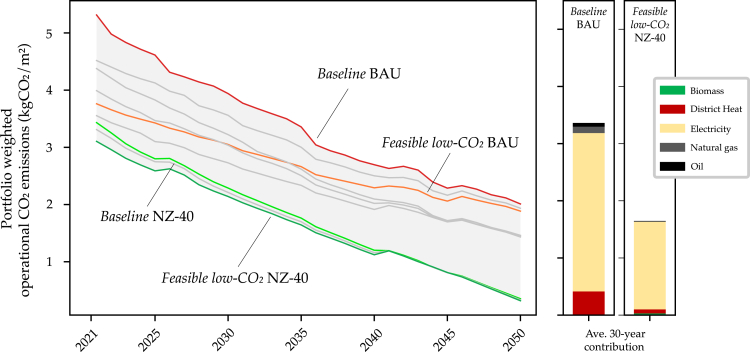
Operational CO_2_ emissions reduction pathways Results presented for the first five years for *Baseline* BAU and *Feasible low-CO*_*2*_ BAU portfolio strategies (RCP 4.5, fleet-level policy implementation).

In Figure 6, we present the operational CO_2_ emission reduction pathways, highlighting the *Baseline* and *Feasible low-CO*_*2*_ strategies for the BAU and NZ-40 scenarios. For all strategies, operational emissions reduce throughout the 2021–2050 time horizon as buildings conduct retrofit interventions on the demand- and supply-sides. Comparatively to the *Baseline* BAU pathway, the *Feasible low-CO*_*2*_ NZ-40 decarbonization pathway demonstrates a cumulative reduction of 51%. The majority of these emission reductions happen early in the horizon (42% difference in 2021) because of deep retrofits.

Operational CO_2_ emissions pathways are highly influenced by the electricity grid decarbonization within each policy scenario (Data S1), key for reducing building sector Scope 2 emissions.^45^ Owing to the overwhelming conversion of over 88% of assets in *Baseline* BAU toward RE-driven heat pumps and biomass boilers in the first five years, Scope 2 emissions greatly outweigh Scope 1. These imminent changes in energy systems mean that the resulting 2021 lifecycle operational CO_2_ emission results are half of the reported GRESB values in Table 3 (*Baseline* BAU: 5.3 kgCO_2_/m^2^, GRESB: 10.25 kgCO_2_/m^2^). Even in the *Baseline* BAU strategy, the impact of thermal electrification can be seen with the “emissions burden” being moved outside of the building. Here, over 82% of operational emissions are from electricity and 5% from fossil fuels, whereas the majority of emissions for the buildings’ today come from fossil combustion (Scope 1).

**Table 3:** Real estate portfolio energy and emissions data from 2020 GRESB reporting

Portfolio	Heating^a^ (kWh/m^2^)	Electricity (kWh/m^2^)	Total end energy (kWh/m^2^)	Scope 1 (kgCO_2_/m^2^)	Scope 2 (kgCO_2_/m^2^)	Scope 1& 2^b^ (kgCO_2_/m^2^)
1	75	30	105	10.0	3.0	13.0
2	50	35	85	6.0	1.5	7.5
3	40	40	80	1.0	2.0	3.0
**Agg.**	**65**	**30**	**95**	**8.0**	**2.25**	**10.25**

a Heating combines Fuels and District Heating categories.

b Note that reported GRESB Scope 1 & 2 values are calculated with different methodologies, likely not life cycle emissions. All values are weighted averages based on floor area. To assure anonymity, values are rounded.

Similar to operational emissions, heating demands cumulatively reduce between 43 and 59% over the horizon (Figure S4). As few envelope retrofits are conducted right away in the *Baseline* BAU strategy, the heating demand pathway results can be more directly benchmarked to the GRESB reported value of 65 kWh/m^2^. Here, the *Baseline* BAU strategy begins in 2021 at 51 kWh/m^2^ and the *Feasible low-CO_2_* NZ-40 strategy at 37 kWh/m^2^. Throughout the horizon, electricity demands increase slightly because of (1) occupancy-based norms governing lighting and plug-loads, and (2) cooling demands increasing because of effects of climate change in RCP 4.5 (*Baseline* BAU: 45 kWh/m^2^, GRESB: 30 kWh/m^2^).

### Low-CO_2_ building technology packages

**Figure 7:**
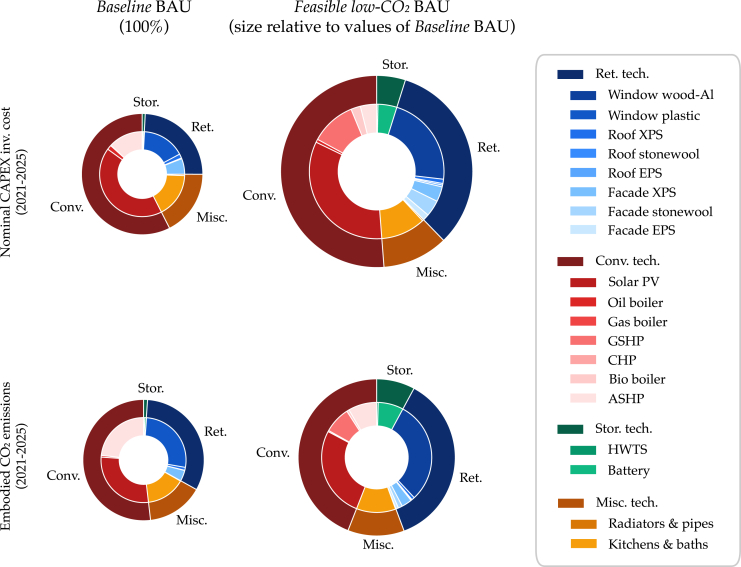
Technology packages’ contribution to CAPEX and embodied emissions Utilizing scenario inputs, the MANGOret optimization model develops optimal strategies (presented as aggregations in the Pareto fronts) for unique building assets. These strategies are fed-in to the portfolio-level model which optimally chooses the best strategies toward its own Pareto front. Depending on the policy alignment approach, the model is constrained differently with regards to performance-based requirements at the building- or fleet-levels.

Figure 7 presents the owners’ near-term CAPEX investments to 2025 for specific technologies and components, with their associated embodied emissions footprints for two points in the BAU policy scenario. In the *Feasible low-CO*_*2*_ BAU strategy, the vast majority of investments are toward solar PV (33%), wood-aluminum windows (22%), and heating systems (18%), accounting toward 27%, 30%, and 17% of embodied emissions respectively. Li-ion battery storage accounts for 5% of investments and contributes to 7% of embodied emissions. Comparatively, the *Baseline* BAU strategy has lower CAPEX and embodied emissions in the first five years because of later investments in window retrofits, solar PV, and heating systems which are pushed toward end-of-life. The 30-year embodied emissions differ by 16% between the strategies (Figure 5).

By modeling the long-term evolutions of (1) energy carrier prices, (2) CO_2_ taxes, (3) technological learning from RE technologies with (4) prospective policy measures, our results demonstrate a more “level playing field” for high-CAPEX, RE-based systems becoming more attractive than incumbent fossil fuel technologies (low-CAPEX, high-OPEX). This aligns with recent studies^40^^,^^61^^,^^62^ indicating a paradigm shift away from fossil fuels toward RE-based and sector-coupled energy systems because of the superior techno-economics.

Specifically, optimal solutions rely heavily on ground- & air-sourced heat pumps (G & ASHPs) with biomass boilers for winter peaks coupled with solar PV and batteries. These low-CO_2_ heating systems account for a minor share of CAPEX and embodied emissions (<10%) in the *Feasible low-CO*_*2*_ BAU strategy. Although many assets have existing oil and gas boilers (assumed to be paid off), even in the *Baseline* BAU strategy these systems are seldom used representing stranded energy systems.

The speed of the paradigm shift is strongly impacted by stringent EE regulations and attractive incentives in the NZ-40 scenario. For example, attractive policies for solar PV such as feed-in-tariffs and incentives result in an investment spike in 2021 even in the *Baseline* BAU strategy. Here, over 42% of CAPEX investments in the first five years are toward solar PV. Furthermore, in the NZ-40 scenario, facade stone wool insulations play a much larger role in CAPEX.

## Discussion

In this study, we optimize portfolio retrofitting strategies toward cost-effective decarbonization to 2050. We explore strategically valuable policies and managerial insights for the transition toward whole-life Net Zero CO_2_ portfolios by considering optimal investments in building assets under various policy conditions, moving away from single policy instrument analyses.^63^^,^^64^ Considering policy mix scenarios shows that for the extreme strategies *Min-cost* BAU and *Min-CO*_*2*_ NZ-40 (fleet-level), whole-life CO_2_ emissions to 2050 can be reduced by 47%, with lifecycle operational CO_2_ reducing by 69%. The range of results in between demonstrates the importance for policymakers from city to national-levels to provide policy certainty to alleviate transition risk.

Owing to their distinctive starting conditions and existing systems, building assets have varied energy and CO_2_ reduction potentials, and therefore costs. Scaling bottom-up asset strategies to portfolio plans, assets lie in a distribution from hard-to-decarbonize to low-hanging-fruit. In this light, we explore a policy innovation to regulate portfolio CO_2_-performance as a building fleet, similar to automotive industry regulations. Moving away from a “one-size-fits-all” approach for performance-based policies on EE and CO_2_ benchmarks could reduce transition costs 8% with equal emissions. Although a fleet perspective means that dirty assets still remain, it could prove important to give owners flexibility to manage the high-cost transition in time to 2050. Such flexibility could also increase acceptance of low-emission building standards and help break political deadlocks.

Decarbonized real estate portfolios require a significant number of deep retrofits earlier-than-planned, comprising complete re-insulation of building envelopes coupled with RE-based heating systems. Significant CAPEX into retrofits in the near-term could create capital liquidity and workforce adequacy issues, such as the burgeoning “green” buildings and construction workforce supported in the upcoming European Renovation Wave.^6^ Owners used to smoothing CAPEX over the years could try to sell (brown-spin) or redevelop assets which are flagged as stranded. This could potentially further grow the carbon bubble in the building sector, necessitating both owner and policymaker urgency.

Operational Scope 1 & 2 CO_2_ emissions can be optimally reduced by over half between extreme strategies. Even in minimum-cost strategies, we show a paradigm shift toward RE-based heating systems comprising heat pumps and biomass boilers coupled with solar PV. Many assets with existing fossil fuel boilers seldom use them because of the techno-economic inferiority, flagging stranded energy systems. Although Scope 1 emissions are low, Scope 2 emissions with regards to electricity grid decarbonization play an important role. On the other hand, embodied Scope 3 CO_2_ emissions hold the largest emissions share in low-CO_2_ strategies (50–60%) and are largely impossible to avoid. There is a need to develop commercially-available low-embodied emission options for the key culprits: insulation materials, window frames, with lowering values for solar PV and batteries. To reach Net Zero targets, significant offsetting will be required.

### Limitations of the study

Our results are subject to several limitations. First, all optimizations conducted in this study are deterministic and assume perfect foresight for all future-looking data. Future work could focus on uncertainty and sensitivity analyses to “stress test” asset and portfolio strategies for various future uncertainties such as climate change risks^65^ and the recent geopolitical developments for European fossil fuels.^66^ Second, we rely on building archetypes and government databases for building asset-level datapoints which are subject to inaccuracies. Third, there is no assessment of the contributions of each individual policy instrument to the overall impact of the policy mix on optimal strategies. Similarly, the fleet-level policy implementation approach needs to be further explored as there is currently no precedent to the knowledge of the authors. Fourth, our work lacks an assessment of the financing mechanisms and distributional aspects of retrofitting real estate assets relating to the landlord-tenant split-incentive. Lastly, our study does not fully consider the potential co-benefits of building sector decarbonization, such as infrastructure resilience, air quality, water quality, and human health, which could improve socio-economic attractiveness.

## STAR★Methods

### Key resources table

**Table:** 

REAGENT or RESOURCE	SOURCE	IDENTIFIER
**Deposited data**		

Archetypal energy demand database	Petkov et al. (2022)^33^	https://doi.org/10.1016/j.apenergy.2022.118901
Policy scenarios and related costs and incentives	This paper and supplementary information	Tables 3 and S2
Techno-economic data	Petkov et al. (2022)^33^	https://doi.org/10.1016/j.apenergy.2022.118901
OpenStreetMap	OpenStreetMap Foundation^67^	https://www.openstreetmap.org/
STATENT	Federal Statistical Office of Switzerland^68^	https://www.bfs.admin.ch/bfs/de/home/statistiken/industrie-dienstleistungen/erhebungen/statent.html
GWR	Federal Statistical Office of Switzerland^69^	https://www.bfs.admin.ch/bfs/en/home/registers/federal-register-buildings-dwellings.html
Renewables.ninja	Pfenninger et al. (2016)^70^ and Staffel et al. (2016)^71^	https://doi.org/10.1016/j.energy.2016.08.060 https://doi.org/10.1016/j.energy.2016.08.068 www.renewables.ninja
MERRA-2	Gelaro et al. (2017)^72^	https://doi.org/10.1175/JCLI-D-16-0758.1

**Software and algorithms**		

Python 3.7.1	Python Software Foundation^73^	https://www.python.org/
Gurobi 9.0.0	Gurobi Optimization LLC^74^	https://www.gurobi.com/

### Resource availability

#### Lead contact

Further information and requests for resources should be directed to and will be fulfilled by the lead contact, Dr. Ivalin Petkov (epetkov@optiml.com).

#### Materials availability

The study did not generate new materials.

### Method details

#### Asset-level optimization framework

We utilize the MANGOret (Multi-stAge eNerGy Optimization — retrofitting) optimization framework and model for the long-term investment planning of existing building retrofits.^33^ The framework approaches the asset-level investment decision-making problem from the disciplines of energy system modeling, building physics, and real estate management. The optimization model is formulated as a Mixed-Integer Linear Program (MILP) in Python using the Pyomo open-source optimization modeling language,^73^^,^^75^ solved with Gurobi.^74^

The framework and model can accommodate any unique building in a scalable manner to develop optimal investment strategies considering a large set of technologies on a life cycle basis. Considering “both sides of the energy balance” in the model formulation allows to consider the interdependencies of technology scheduling, for example, how the timing and design of an envelope retrofit would impact the heating system design and operation.

The demand-side comprises thermal envelope energy efficiency with over 10 retrofitting packages for Facade, Roof, and Windows, consisting of various technology options which vary on a cost vs. embodied emissions basis. Namely, these are various options for insulation materials: oil-based extruded and expanded polystyrene (XPS and EPS, respectively) along with mineral stonewool, and windows: plastic and wood-Aluminum frames. These retrofitting technologies also have a depth element — the minimum Building Energy Code or target green building label (e.g. LEED, BREEAM, DGNB, Minergie, etc.).

The supply-side comprises energy supply, conversion, and storage, modeled in the popularized Decentralized Multi-Energy Systems (D-MES) framework considering multiple energy carriers: heating, electricity, natural gas, biomass, oil, and District Heating (DH).^76^^,^^77^ The candidate technologies include: electrically-driven Air-Source Heat Pumps (ASHP), Ground-Source Heat Pumps (GSHP), fuel oil, natural gas, and biomass boilers, gas-fired Combined Heat and Power (CHP) engines, and DH. In terms of renewable energy technologies, only solar Photovoltaic (PV) panels are considered due to urban constraints. Additionally, Hot Water Thermal Storage Tanks (HWTS) and lithium-ion batteries are considered to store thermal and electrical energy, respectively. Buildings can import all energy carriers but can only export electricity as they are grid connected. Existing technologies (e.g. boilers) and DH connections for each building are also included, based on data availability. Non-energy components critical for real estate owners’ retrofitting budgets are also included, namely: kitchens, bathrooms, and piping. To provide better accuracy for intervention timing, the model considers the component condition degradation utilizing the Schroeder method.^78^

This study considers three real estate portfolios with all building situated in Switzerland. While we leverage datasets existing for Switzerland, a similar model approach can be used in other European countries with similarly available open-source data relevant for building retrofitting.

The MANGOret framework^33^ requires a small set of building-specific data from real estate owners: address, construction year, renovation year, and last year’s energy demands. Other techno-economic (e.g. technology CAPEX learning curves and energy carrier price evolutions) and environmental parameters (e.g. Electricity grid decarbonization and CO_2_ tax evolution per policy scenario) with long-term projections are all given in the original model formulation and visually presented in the Data S1. MANGOret is a deterministic optimization framework which advances beyond the traditionally single-stage building energy optimization models using approaches such as life-cycle costing.^79^ Other methodologies have recently been explored to make building energy decision-making problems more tractable such as artificial neural networks based on machine learning techniques.^80^^,^^81^

We leverage an archetypal energy demand database to reference demands of various retrofitting packages for many unique buildings. The database consists of over 2,100 Swiss archetypes varied by building type, geographic zone, and age categories, which were simulated for from 2020–2060 in 10-year time-steps for 3 climate change scenarios based on two RCPs: 4.5°C, and 8.5°C. We utilize the CESAR (Combined Energy Simulation And Retrofitting) tool^82^ built on the standard building energy software EnergyPlus.^83^ The archetypes were developed based on OpenStreetMap^67^ and government building statistics databases.^68^^,^^69^ Solar irradiance data for each building location are taken from the Renewables.ninja API which connects to the MERRA-2 database.^70^^,^^71^^,^^72^ We cluster the energy demand and solar irradiance time-series parameters using the k-medoids peak + typical day clustering approach,^84^ in this study for two peak days for electricity and heating demand, and five typical ‘normal’ days, resulting in seven total typical days.

We optimize each asset location *A*_*l*_ on a multi-objective basis, minimizing cost and CO_2_, for each year *y* for the entire time horizon 2021-2050. The multi-objective optimization outputs the Pareto front, consisting of seven points. Seven Pareto points are chosen as typical from other optimization studies to present sufficient strategic options (five) between the two objective extremes (minimum-cost and minimum-emissions) for the portfolio-level optimization.

The cost function comprises costs *C* and revenues *R*. CAPEX investments and salvage values are considered for all technologies and components. OPEX considers maintenance along with energy imports and exports. We refer the reader to the original mathematical formulation for more descriptive details.^33^

$$\underset{Asset\;total\;cost}{\underset{︸}{\min\;A_{l}^{cost}}}=\underset{CAPEX}{\underset{︸}{\sum_{y}\left(\underset{Energy\;systems}{\underset{︸}{C_{l,y}^{INV,TECH}}}+\underset{Envelope}{\underset{︸}{C_{l,y}^{INV,RET}}}+\underset{Misc.}{\underset{︸}{C_{l,y}^{INV,MISC}}}+\right)-\underset{Salvage\;value}{\underset{︸}{R_{l}^{SLVG}}}}}+\sum_{y}\left(\underset{OPEX}{\underset{︸}{C_{l,y}^{IMP}+C_{l,y}^{MAINT}-R_{l,y}^{\mathrm{EXP}}}}\right)$$

The emissions function comprises emissions *E* for embodied emissions of all technologies and components relevant for existing building retrofits (Scope 3) along with operational emissions within the building (Scope 1) and indirect emissions from energy imports (Scope 2).

$$\underset{Asset\;total\;{CO}_{2}}{\underset{︸}{\min\;A_{l}^{{CO}_{2}}}}=\sum_{y}\left(\underset{Embodied\;{CO}_{2}}{\underset{︸}{\underset{Energy\;systems}{\underset{︸}{E_{l,y}^{{embCO}_{2}TECH}}}+\underset{Envelope}{\underset{︸}{E_{l,y}^{{embCO}_{2}RET}}}+\underset{Misc.}{\underset{︸}{E_{l,y}^{{embCO}_{2}MISC}}}}}+\underset{Oper.\;and\;indirect\;{CO}_{2}}{\underset{︸}{E_{l,y}^{{operCO}_{2}}}}\right)$$

While operating emissions can be easily determined and accounted for based on the energy consumption of the assets in the portfolio, accounting for embodied emissions is not as clearly defined. In our model, the embodied emissions of a technology are assigned to the year in which the technology is installed. We choose this accounting approach because we argue that on a physical basis, the CO_2_ emissions embodied in materials and technologies were already released into the atmosphere by the time of installation in the building. Therefore, spreading these embodied emissions evenly over the component lifetime, as done in Switzerland,^85^ or using time-dependent weighting factors (e.g. emissions released now have a greater impact than those at the end-of-life) as done in the French RE2020,^86^ is a non-physical accounting measure which does not address the urgency of decarbonization.

The asset value is calculated with the industry-accepted Discounted Cash Flow (DCF) methodology: rental revenues less the costs. Rental revenues are calculated based on value-added investment formulated based on legally-mandated rental calculations.

$$\underset{Asset\;value}{\underset{︸}{A_{l}^{value}}}=\underset{Discontinue\;cash\;flow}{\underset{︸}{\left(\sum_{y}{rent}_{l,y}\right)-A_{l}^{cost}}}$$

#### Portfolio-level optimization

We replicate the decision-making approach of the real estate multi-year planning process within the MANGOret framework. We do so by nesting the asset-level optimization within the portfolio-level optimization in a two-step approach. First, as previously described, we optimize each individual asset on a multi-objective cost and CO_2_ basis.^33^ Here, we formulate a smaller portfolio-level model. Based on the seven optimal Pareto points *pp* for each asset *A*_*l*_, the portfolio optimization conducts the same multi-objective optimization, minimizing cost and CO_2_, by choosing the optimal asset-level strategies for the portfolio-level planning. It does so by utilizing a binary variable, $Y_{l,pp}^{sol}$, which references the chosen asset strategies total cost, CO_2_, and value.

$$\underset{\mathrm{Portfolio cost}}{\underset{︸}{\min\;P^{cost}}}=\underset{\mathrm{Chosen asset strategy}}{\underset{︸}{\sum_{l,pp}\left(Y_{l,pp}^{sol}\cdot A_{l,pp}^{cost}\right)}}$$

$$\underset{\mathrm{Portfolio}\;{\mathrm{CO}}_{2}}{\underset{︸}{\min\;P^{{CO}_{2}}}}=\underset{\mathrm{Chosen asset strategy}}{\underset{︸}{\sum_{l,pp}\left(Y_{l,pp}^{sol}\cdot A_{l,pp}^{{CO}_{2}}\right)}}$$

$$\underset{Portfolio\;value}{\underset{︸}{P^{value}}}=\underset{\mathrm{Chosen asset strategy}}{\underset{︸}{\sum_{l,pp}\left(Y_{l,pp}^{sol}\cdot A_{l,pp}^{\mathrm{value}}\right)}}$$

Only one Pareto point can be chosen per asset. The chosen solutions at the portfolio-level must be below the available managerial resources of the owner, to limit the projects per year. The chosen solutions at the portfolio-level must be below the portfolio’s total CO_2_ emissions target.

$$\sum_{pp}Y_{l,pp}^{sol}=1\text{,}$$

∀l∈L

$$\sum_{pp}Y_{l,pp}^{sol}\cdot Y_{l,pp,y}^{retoccur}\leq M\text{,}$$

∀y∈Y

$$\sum_{l,pp}Y_{l,pp}^{sol}\cdot A_{l,pp}^{{CO}_{2}}\leq {CO}_{2}$$

#### Policy scenarios

To advise investors and policymakers on how to intervene under future uncertainty, the focus of consideration needs to be broadened through the use of scenarios, as suggested by Moss et al.,^87^ Swart et al.,^88^ and many others. Scenarios are alternative images of how the future might unfold and a set of scenarios assists in the understanding of possible future developments of complex systems.^2^ According to Grant et al.^48^ an appropriate reference scenario must be taken into account to compare impacts in the context of policies and technological change.^2^

Following their logic, we develop three comprehensive building sector policy mix scenarios encompassing over ten instruments from different policy domains. The three scenarios represent: (i) a *policy stagnation* reference scenario (BAU – *Business-as-usual*), (ii) a *current ambition* scenario (NZ-50 - *Net Zero 2050*), and (iii) a *maximum ambition* scenario (NZ-40 - *Net Zero 2040*) to ensure the findings are relevant to the Swiss and European energy policy context. Historical narratives of the individual policy instruments provided the background for the policy mix scenario development in the following four steps:

1. Setting of distinct 2050 and intermediary emissions goals using dual forecasting and backcasting approaches, as recommended by Kishita et al.^49^
2. Developing specific policy measures for each scenario targeting their emission goals, focusing on regulatory, market-based, and financial incentive instruments.
3. Validation of the scenarios in an expert elicitation workshop with 14 experts from real estate owners, industry stakeholders, and policymakers.
4. Verification of internal consistency & correlation matrix (Table S1) and quantification of the specific policy instrument values (Table S2).

Three distinct emission goals to be achieved by 2050 are qualitatively defined and set the particular desired future end-point for the individual scenarios. We use the national Swiss Energy Strategy 2050^11^^,^^89^ (SES 2050) as the background policy framework.

1. **BAU (*Business-as-usual*)** reflects the 2020 status quo with a focus on the depth of retrofitting regulations without incentivizing speed. The Net Zero 2050 target is not met.
2. **NZ-50 (*Net Zero 2050*)** represents the official SES 2050 policy objectives through accelerated retrofitting with relevant regulations to meet intermediate goals to Net Zero 2050.
3. **NZ-40 (*Net Zero 2040*)** represents maximum ambition interventions to the existing retrofitting-relevant regulations, with the goal to meet the Net Zero 2050 emission target as early as 2040.

Table 2 presents a qualitative overview of the individual policy instrument setting for the three scenarios outlined above.

#### Policy implementation approaches

We explore two performance-based policy implementation approaches comprising the following instruments: thermal EE, CO_2_ performance, renewable heating, and on-site electricity production requirements. These can be enforced at either the (i) building asset or (ii) fleet-level and are visually presented in Figure 1. Currently, BEC regulations are enforced as benchmarks (e.g. minimum energy efficiency of 36 kWh/m^2^upon retrofit) at the building-level, varying by building type.

Moving towards a fleet-level approach regulated by the same benchmarks but as a weighted average value for the entire portfolio provides more investment flexibility. As the horizon of our model is 2021-2050, we implement performance-based policy instruments as 30-year average values. When a hard-to-decarbonize asset cannot reach a stringent benchmark, the optimal minimum-emissions strategy is chosen. In future work, policy instruments could be implemented as step-changes in the long-term horizon model.

#### Key assumptions

We convert cooling demand to electrical demand. If a building has an existing DH connection, no gas connection is allowed. DH is assumed to be waste-powered. Discount rate: 5%.

## Data Availability

• The sources of the datasets supporting the current study are presented in the method details section - ‘‘Policy implementations” and the supplementary information section “Policy scenarios”. Relevant data and codes can be available on request from the lead contact. • This paper does not report original code. • Any additional information required to reanalyze the data reported in this paper or reproduce the results is available from the lead contact upon request.

## Supplemental information

Document S1. Figures S1–S6

Data S1. Extended data for making the figures in the main text
