# Supplementary material for: Decarbonizing real estate portfolios considering optimal retrofit investment and policy conditions to 2050
Source: iScience. 2023 Apr 8;26(5):106619. doi: 10.1016/j.isci.2023.106619 (PMC10165412; doi:10.1016/j.isci.2023.106619)
Supplement: Document S1. Figures S1–S6 [file mmc1.pdf]

iScience, Volume *26*

Supplemental information

Decarbonizing real estate portfolios considering optimal retrofit investment and policy conditions to 2050

Ivalin Petkov, Alicia Lerbinger, Georgios Mavromatidis, Christof Knoeri, and Volker H. Hoffmann

Decarbonizing real estate portfolios considering optimal retrofit investment and policy conditions to 2050 – Supplementary Information

Ivalin Petkov[1,2,], Alicia Lerbinger[1], Georgios Mavromatidis[1], Christof Knoeri[1], Volker H. Hoffmann[1]*

[1] *Group for Sustainability and Technology, ETH Zurich, 8092 Zurich, Switzerland*

[2] *OPTIML AG, 8003 Zurich, Switzerland*

* Corresponding Author and Lead Contact: e-mail: ivalin.petkov@alumni.ethz.ch

Supplementary Material Table of Contents

Decarbonizing real estate portfolios considering optimal retrofit investment and policy conditions to 2050 – Supplementary Information 1

Supplementary Material Table of Contents 1

Policy scenario methodology..... 2

Policy scenario framework and historical narratives..... 2

Policy scenario development..... 2

Expert elicitation workshop and interviews 3

Policy scenarios 5

Policy scenario framework and historical narratives..... 5

Policy scenario development..... 9

Expert elicitation workshop and interviews 14

Policy instrument evolutions and model implementation..... 15

Supplementary results..... 20

References..... 23

Policy scenario methodology

Here we describe the policy scenarios in more detail. First, we describe the policy scenario framework and historical narratives, followed by the policy scenario development with granularity towards individual policy instruments.

Policy scenario framework and historical narratives

Frameworks are needed to contextualize scenarios to assess the implications of future policy changes on building retrofitting investments[1]. The framework for the building sector policy scenarios is based on both intergovernmental and national strategies, consisting of the IPCC SRES (Intergovernmental Panel on Climate Change - Special Report on Emissions Scenarios)[2], the SES-2050 (Swiss Energy Strategy 2050)[3], along with the European Renovation Wave[4,5] and Energy Performance of Buildings Directive (EPBD)[6].

While high-level Swiss and European climate policies are based on the Paris Agreement[7] goals, which themselves are based on the first IPCC assessment report from 1990[8] and the Kyoto Protocol from 1997[9], they do not provide specific details on policy measures for the building sector. In order to elaborate on future climate risks, academic and industry studies have developed policy scenarios with higher levels of detail relevant for the building sector. This has necessitated constructing scenario narratives which encapsulate the most relevant policy instruments such as Building Energy Codes (BECs), climate regulations, incentives, along with tenant as well as real estate market regulations.

Narratives give descriptive guidance on constructing scenarios and act as the projection boundary for each key instrument, leading to a broader perspective[10,11]. To understand and describe the steady-state Swiss and European building sector policy context, a detailed description of historical and current laws and norms, relating to building stock decarbonization and retrofitting, is provided. Regulations, incentives, and market aspects are discussed based on a literature review. Simultaneously, the policy instrument toolkit is built from the narratives.

Policy scenario development

In total, three policy scenarios are developed, each of them characterizing dissimilar emission pathways. The scenarios are derived in four steps:

First, distinct 2050 emissions goals are set using dual forecasting and backcasting approaches, as recommended by Kishita et al.[12], to provide a reference basis for future scenarios. These approaches seem to be appropriate for envisioning futures and identifying long-term risks whilst identifying initial conditions, drivers of change, and the bandwidth of initial trajectories towards long-term sustainability goals[11].

On the one hand, forecasting approaches are known to be predictive, defining the present as the starting point. The upside of forecasting is that one can draw several transition paths to the future. However, a direct connection between the present status and a vision is not necessarily given. The IPCC SRES scenarios are an example of forecasting scenarios.

On the other hand, backcasting approaches try to explore possible paths backwards from predetermined starting points in the future to the present[13]. Thus, the direct connection of the particular desired future end-point with the present is guaranteed, and the feasibility of predetermined visions derived from a backcasting approach should be ensured despite various future uncertainties[12]. Furthermore, backcasting approaches allow the exploration of futures regardless of fundamental uncertainty, resulting in entirely new scenario explorations.

Second, once the intermediate and the 2050 emission goals for the three scenarios are defined, specific policy measures are developed for each scenario. The simultaneously to the narratives developed policy toolkit, focusing on regulatory, market-based, and financial incentive instruments, builds the basis for this step.

Third, as the characteristics of the building stock are highly dependent on different trends related to socio- and macro-economic factors, forecasts should reflect as much as possible the current economic and social circumstances[14]. Thus, the long-term context parameters and future trends of the real estate market development, interest rates, component and energy prices are assumed based on probability distributions. Context parameter developments are aligned with other macro scenarios such as the 'Business as usual' (WWB), 'New energy policy' (NEP) and 'Political measures' (POM) scenarios in the SES-2050[3].

The fourth and last step involves the verification of internal consistency and the alignment with the historical narratives. Finally, the three scenarios are cross-checked on disparities and their distinctiveness in a correlation matrix presented in the Supplementary Table 1.

Expert elicitation workshop and interviews

Proceeding from qualitative scenario narratives to quantitative-projection scenarios is seen as one of the most challenging issues in constructing and applying scenarios in models[10]. Hence, the developed scenarios are validated through an expert elicitation workshop to decide upon the most relevant regulatory instruments for evaluating future building retrofitting investments. While such an expert panel provides beneficial in-depth knowledge of the various stakeholders on the scenario net effects, Woodward and Bishop discuss this method as a problem of choice under pure uncertainty[15]. An expert will always assign probabilities on different scenario outcomes based on the maximization of their subjective expected utility. Thus, the experts cannot be expected to arrive at a consensus about the accuracy of their suggested probabilities. Such a panel discussion is rather beneficial to obtain diversified

opinions. Therefore, the workshop aims to receive feedback from the experts on the proposed scenarios to refine and quantify the regulatory measures for the modeling.

The group of 14 participants consisted of real estate owners from different sectors (e.g. banks, pensions, insurances, and public authorities), industry, and regulatory experts. The workshop took place in early October 2020. The majority of the experts assembled physically in Zurich, Switzerland, while some joined the workshop online via Zoom. The participants received the scenario narratives and a summary table with the policy toolkit trends a few days before the meeting, allowing them to familiarize themselves with the workshop content in advance.

At the beginning of the workshop, the experts were asked to give their opinion on the completeness of the used policy instrument toolkit. The focus was on complementing, from their perspective, other vital instruments. This short intro was followed by the main scope of the workshop: The experts were divided into three groups and asked to discuss and complete the prepared worksheet. A total of four questions were asked to answer and discuss:

i. Are the scenario narratives distinct from each other? If not, what could be improved?
ii. Are the considered policy instruments in the toolkit comprehensive? Should some be added or removed?
iii. Do you agree with the policy instrument trends (arrows) within each scenario?
iv. Are the trends within each scenario internally consistent (i.e. do some conflict with each other)?

Questions (ii) and (iii) are designed to validate the prior developed scenarios and quantify the proposed level and timing of regulatory measures. Questions (i) and (iv) aim to compensate for any inconsistencies and adjust the scenarios to each other. At the end of the workshop, the main findings of the three groups were discussed in the plenum.

In addition to the workshop, three semi-structured interviews were conducted in order to justify the developed policy scenarios. The expert group consisted of two national and cantonal regulators and one building valuation expert. This mix of interviewees allowed the focus on incorporating aspects from the relevant sector, subsector, and roles to cover the different perspectives in the interviews. The main interview questions can be categorized in the following:

i. Describing the current retrofitting landscape with focus on the cantonal energy laws, MuKEn 2014[16], incentive programs as well as existing regulatory tensions and barriers towards retrofitting the building stock.
ii. How to cooperate with building owners of various sizes on sustainability issues?
iii. How do regulatory instruments differ depending on building or ownership type?
iv. Opinion on future policy scenarios relevant for retrofitting buildings and district levels.
v. How do standard valuation methods incorporate retrofitting strategies and how are uncertainties reflected in the process?

Each interview lasted about one hour and was audio-recorded (for both in-person and phone interviews), to enable full transcription for scientific accuracy and ethics.

Policy scenarios

In the following, the contextualizing frameworks and historical narratives, the scenarios, and the policy instrument developments based on the expert elicitation workshop are described. The policy scenarios are input into the modeling framework in different ways depending on the instrument in question, discussed in the following.

Policy scenario framework and historical narratives

The contextualizing frameworks (i.e. SRES and SES-2050) are intended to provide consistency throughout the elaborated policy scenarios. The SRES report draws four narrative storylines, covering two different future market developments (globalization and sustainability), to add context for the scenario quantification. The four presented storylines describe different demographic, social, economic, technological, and environmental developments, covering a wide range of driving forces contributing to GHG emissions[2].

Analogous to this, model-based scenarios are presented in the context of the SES-2050. These scenarios, specifically designed for the Swiss context, act as a basis for decision-making processes[17]. The SES-2050 attempts to demonstrate and compare the effects of framework conditions, influencing factors, political interventions as well as objectives on energy consumption, CO_2 emissions and supply security, through the use of the scenarios.

Analyzed policy instruments relevant to retrofitting such as BECs, labels, certificates, financial regulations, incentives and subventions along with tenant and climate laws, and their combinations (i.e. policy mixes) are disparate across sectors. Thus, the investigation mainly focuses on high-level Swiss and European policy instruments. Hereafter, (i) fundamental climate-related agreements, to which the Swiss Federal Council has committed itself, are described and followed by a brief description of the implementation of these conventions in Swiss regulations. Further, (ii) an in-depth look at the Swiss BECs and regulations is taken. Lastly, (iii) the focus is set on the Swiss tenancy law, which significantly influences and regulates the execution and the associated economic viability.

Climate-related agreements and laws

Switzerland signed the Kyoto Protocol in 1997, setting the legal framework for the CO_2 law enacted in 2000[18]. The CO_2 law defines a GHG reduction pathway towards a final goal of 20% domestic GHG emissions reductions by 2020 (from 1990 levels)[19]. To monitor the success of the measures introduced, intermediate targets were individually defined for the building, transport and industry sectors[20]. The latest intermediate target for the building sector is to

reduce CO_2 emissions by at least 40% below 1990 levels. To achieve these building sector intermediate targets, it is very relevant to reduce the heating and process fuel emissions (i.e. heating oil, natural gas, wood, and coal)[19]. Consequently, the Council enacted a CO_2 tax by 2008 to stimulate a reduction in heating and process fuel consumption. Emissions of other fuels used in the transport sector (i.e. gasoline and diesel) have different intermediate targets[21]. The CO_2 law intends to increase the tax rate for the coming years if the intermediate targets are not met. The extent, to which the tax rate is increased, depends on the deviation from the target value[22]. Ever since its introduction, the Council increased the CO_2 tax with more stringent intermediate goals. As of today, the levy on CO_2 emissions is at 120 CHF/tonCO_2 – recently increased from 96 CHF/tonCO_2[21]. In 2022, EUR and CHF exchanged at nearly similar rates).

In 2016, the Energy Law (Energiegesetz EnG) was ratified with the intention to secure an economic and environmentally-friendly energy supply[22]. This law, paired with the revised CO_2 law from 2013, enabled the political framework for the Paris Agreement-based SES-2050, enacted into force in 2018. The SES-2050 set into law the goal to reduce overall CO_2 emissions based on the Kyoto goals, focusing on three main strategic objectives: (i) increasing energy efficiency, (ii) increasing the use of renewable energy, and (iii) withdrawal from nuclear energy. The strategy was further revised in 2019 with the Net-Zero law, targeting a 95% CO_2 emissions reduction by 2050.

BECs and regulations

The history of Swiss BECs and regulations is highly partitioned between national, cantonal, and institutional levels. While a detailed history and temporal development is described in the extended case studies of Schwarz et al., the most relevant developments relating to retrofit-specific regulations today and into the future are described here[23].

The primary Swiss institution in setting building standards is the Swiss Society of Engineers and Architects (Schweizerischer Ingenieur- und Architektenverein - SIA). SIA is a private association which gained a prominent role in setting norms and standards for the Swiss building industry after the failure to enforce a national building energy law in 1983. The Cantons transferred SIA's norms into BECs through the Conference of Cantonal Energy Directors (EnDK), which is still the prevailing model for enforcing cantonal BECs today. The EnDK introduced the first regulation in 1992 with the Rational Energy Use in Buildings (Rationelle Energienutzung in Hochbauten), which has been revised into the MuKEn in 2000[16].

Since then, the SIA introduced various regulations relevant for the construction sector. However, in the context of this work, the SIA 380/1 norm is of importance. Its purpose is to regulate the moderate and economical use of energy for space heating in buildings[24]. The content consists, on the one hand, of construction limit and target (performance) requirements, and on the other hand, of principles on how to perform heating requirement calculations for any building type. The first version of the norm was enforced in 1992, being

revised and adapted to the state of technology in the years 2001, 2007, 2009 and 2016. The latest revision in 2016 was done in close cooperation with the authors of MuKEn 2014[16]. Both regulations pursue similar objectives, and thus contradictions were resolved accordingly[25].

The MuKEn initiative is part of the SES-2050 and has the primary goal of reducing per capita energy consumption and GHG emissions. It focuses on the implementation and standardization of comprehensive building requirements into the cantonal laws. To ensure flexibility for the Cantons, due to specific circumstances (e.g. high proportion of holiday homes), the MuKEn acts as recommendations from EnDK to be implemented in each Canton through political processes, and thus changes can be adapted. However, these individually adapted cantonal BECs pose a major challenge in the Swiss building sector. The regulations are divided into individual, distinct sections: (i) Parts B - D, J - L, N, and O of the basic module contain the minimum Federal legal requirements for the Cantons (Art. 45 EnG), Parts E - I, M, and P contain the requirements in accordance with the energy policy guidelines of the EnDK, Part N introduces the uniform building Energy Performance Certificate (EPC) of the Cantons (Gebäudeenergieausweis der Kantone GEAK) for whole Switzerland. The additional modules 2 - 11 provide additional requirements for specific basic modules.

Since its enforcement, the MuKEn has been revised twice, in 2008 and 2014, with the recent implementation still ongoing in the Cantons[26]. A further revision is planned for 2025. Central elements of the MuKEn 2014 update are the adaption of the minimum energy performance metrics kWh/m² per building type, which are based on the 2009 updated SIA 380/1 norm. Furthermore, the 2014 update integrates aspects such as on-site electricity production for new buildings (Part E) and renewable heat generation (Part F). Part E requires at least 10 W/m² electricity self-production, but never more than 30 kW of on-site electricity generation taking into account the available roof space. To foster renewable technologies in the building sector, Part F requires a minimum renewable heat production level of 10% in case of fossil fuel boiler replacements. By introducing these mandates in such a manner, the law intends to avoid an implementation of politically unfavorable technology bans (e.g. oil boilers) or mandates (e.g. solar PV). For example, the 10% mandate would require self-production of renewable energy, necessitating a solar PV installation, which in turn makes a heat pump installation more economically attractive than an oil boiler replacement. An introduction of a minimum CO_2 performance (e.g. $kgCO_2/m^2$) was discussed but not included in the 2014 update.

On a national level, a few other labels exist, which are worth mentioning due to their widespread use in the Swiss building sector. First, there is a private label called (Minergie} adopted in 2001, covering general building sustainability aspects, including energy efficiency, livability, etc. The label acts as the main driver of Swiss buildings' energy efficiency[23]. The GEAK label aligns with the EPCs mandated by the EU's Energy Performance of Buildings Directive (EPBD)[27,28], while the Minergie standards commit to a uniform Swiss system among the different cantons aligning to international labels such as LEED, DGNB, and BREEAM. Schmid et al.[29] observe that these private BECs (i.e. Minergie) set stringency benchmarks and thus induce

delayed effects on the stringency of public regulations through the mechanism of technological change.

There is generally an observed trade-off between retrofit speed and depth between regulations. France, for example, holds one of the strictest European retrofitting obligations through the mandate of the retrofit of all private residences consuming more than 330 kWh/m^2/annum (lowest EPC levels – F and G) by 2025, while further blocking the sale of social housing above this threshold. Denmark focuses, similar to Switzerland, on banning the replacement of fossil fuel heating technologies (i.e. oil or gas boiler) upon retrofit initiation, if approved options such as district heating are available. Moreover, generally all European BECs for retrofitting contain prescriptive requirements (e.g. U-value for envelope efficiency) set at lower thresholds than for new construction.

To promote the retrofit rate and to align with requirements or beyond (labels), the individual European countries offer financial incentives, in the form of tax credits, upfront subsidies or other financial instruments. In Switzerland, authorities launched with the Building Program (Gebäudeprogramm) as the key incentive program to promote retrofitting towards Net-Zero. The Swiss federal government finances grants for this program with the CO_2 tax revenues on heating and process fuels[30,31]. One-third of these tax revenues are directly dedicated to the Building Program (max. 450 mCHF). The remaining two-thirds are annually redistributed in the form of global contributions to cantonal technology fonds (max. 25 mCHF), and proportional and uniform to the population and employees[32]. Uniform financial contributions are distributed to the Cantons, allowing the cantonal authorities to grant retrofitting subsidies through individual cantonal subsidy programs.

Tenant law

Swiss tenant laws are regulated on a national level through the Swiss Tenant law (Miete und Pacht von Wohn- und Geschäftsräumen VMWG). The historical progression shows several revisions regulating rental abuses, retrofit and renovation initiation, redevelopment obligations, along with monetary aspects such as reference interest rates, component pass-on rates, and rent controls.

A key relevant article of this law is Art. 14 VMWG (Additional services of the landlord), defining value-enhancing investments and the extent to which these property improvements can be passed on to the tenant in the form of rent increases[33]. According to this article, the building owner has to prove the value increasing share of every investment in order to enforce net rent increases. However, this has proven not to be simple, as evidenced by the numerous rental legal disputes that have been dealt with in arbitration authorities or courts. For an investor or building owner, it is crucial to know whether they can enforce higher expected profitability of the property through increased rents after a retrofit. Therefore, this law considerably affects the retrofit decision-making of an economic-driven investor.

In recent years, the legislation allowed for pass-on rates of 50-70% for value-adding retrofits[34], but this has recently been corrected to lower values due to legal precedents[35,36]. Further, there exist legal uncertainties due to the contestability of initial rents after major retrofits. Under certain conditions, such as housing shortage in the local market or exceeding local rent levels, the increased rents are hardly justifiable[37]. Many of these aspects can be changed on a cantonal level based on political developments. The Swiss constitution regulates the social objective of ensuring the basic need for housing throughout Switzerland[38]. The Confederation and the Cantons are given various competencies, such as to enact protective regulations under tenancy law (Art. 109 BV)[39]. Consequently, the Canton of Geneva enacted the "Loi sur les démolitions, transformations et rénovations de maisons d'habitation (LDTR)" (Residential Demolition, Alteration and Renovation Act), which restricts the demolition of the scarce available housing space by promoting maintenance and refurbishment and controlling the rent levels of retrofitted assets[40]. Similarly, the Canton of Basel City introduced the similar "Wohnraumfördergesetz (WRFG)" (Housing Promotion Act), promoting the preservation of existing affordable housing and providing adequate housing under acceptable conditions.

Another major problem from the investors perspective is the legal aspect of the OR Art. 272 (Swiss law of Obligations). To ensure deep retrofits, the tenants are given an early eviction notice, allowing them enough time to relocate. OR Art. 272 authorizes tenants to extend their tenancy in due time[41]. Thereby, the tenancy can be extended to a maximum of four years for residential properties and up to six years for commercial[42]. However, according to current practice, the legally possible maximum extension period is rarely granted. The approximate extension period is usually between 2-3 years.

It should be noted that a framework rental agreement is enforced in the French-speaking Cantons of Switzerland, applicable to rental agreements for residential premises[43]. The law does not apply to Single-Family Houses (SFH) or luxury apartments with more than six rooms as well as vacation apartments rented for a maximum of three months per year. The framework generally promotes a culture of negotiation based on equality in the rental sector. The existing legislation is clarified to avoid disputes between landlords and tenants[44].

Policy scenario development

Based on the given frameworks and the findings of the historical narratives, three distinct policy scenarios are developed. These scenarios are not based on likelihood or measured, but simply characterize rough future trends in order reveal the relative policy implications of alternative futures[45]. Three distinct emission goals to be achieved by year 2050 are qualitatively defined and set the particular desired future end-point for the individual scenarios.

i. **BAU (*Business-as-usual*)** reflects the 2020 status quo with a focus on the depth of retrofitting regulations without incentivizing speed. The Net-Zero 2050 target is not met.

ii. **NZ-50 (*Net-Zero 2050*)** represents the official SES-2050 policy objectives through accelerated retrofitting with relevant regulations in order to meet intermediate goals to Net-Zero 2050.

iii. **NZ-40 (*Net-Zero 2040*)** represents maximum ambition interventions to the existing retrofitting-relevant regulations, meeting the Net-Zero 2050 emission target as early as 2040.

Each scenario is supplemented with qualitatively characterized policy measures using the developed policy toolkit from the historical narratives previously discussed. The available policy toolkit includes regulatory, market-based, as well as financial incentives, and fiscal instruments. Table 3 in the manuscript provides a comparative overview of the qualitatively defined instrument trends per scenario.

The following text boxes contain the written narratives on the qualitatively developed policy scenarios.

BAU (*Business-as-usual*)

By and large, this scenario keeps the relevant MuKEn 2014 depth metrics for existing buildings. Maximum energy consumption per building type and renewable heat production requirements are kept at constant levels, while electricity self-production is not mandated for retrofits.

Albeit, due to the failure of meeting intermediate emissions targets, the CO_2 tax constant at 120 CHF/tonCO_2. Thus, the long-term continuation of the Building Program after 2025 is guaranteed by the constant available tax revenue. Through the available budget and increasing number of applications for the Building Program, we assume that the granted incentives per component remain constant.

Due to stagnating political realities, the current zoning law densification incentives, component pass-on rates, and eviction notice periods do not exhibit significant changes. Rental control initiatives continue to be passed in urban centers, forcing municipalities towards a regulated approach in support tenants' concerns about affordable housing. Overall, it is likely that the retrofitting rate stays similar at the current level of approximately 1%. Due to the stagnation in almost all segments, the Net-Zero target will not be met by 2050.

NZ-50 (*Net-Zero 2050*)

New legal frameworks for MuKEn focus on both depth and speed through a mix of different metrics, as explicit technology bans (e.g. oil boilers) or mandates (e.g. decarbonized heating) remain politically unfavorable. MuKEn maximum energy consumption requirements undergo a slight derating to bring new and existing building rates closer together, whilst increasing renewable heat production requirements for retrofits. Further, MuKEn introduces light retrofit mandates for inefficient and highly emitting buildings based on performance metrics in order to meet the climate goals. Simultaneously, electricity self-production is also mandated for retrofits.

The CO_2 tax has an increased trajectory, as emission goals are not met. This increases the budget for the Building Program after year 2025, leading to increased available budgets for incentivizing retrofit components and integrated projects (i.e. efficiency and renewable) to promote depth. In addition, electricity self-consumption is incentivized. In order to further promote the speed of integrated projects, a supplementary incentive program is introduced by 2035 to increase the retrofit rate for retrofits skipping at least two GEAK categories. Cantonal zoning laws follow a similar path with densification incentives coupled to achieved GEAK levels. Consequently, the overall demand for voluntary building labels is increasing.

Tenant laws, including component pass-on rates (increased) and eviction notice periods (decreased), are slightly relaxed in the interest of the property owners. All of these factors lead to an increasing retrofitting rate in urban centers, forcing municipalities to shift political and budgetary attention to support lower-income renters for affordable housing. The retrofitting rate stagnates in rural areas due to decreased potential for rent increases in the market.

NZ-40 (*Net-Zero 2040*)

Analogous to NZ-50, both depth and speed metrics are enhanced with the goal of achieving the full technical maximization of energy and CO_2 metrics for all buildings in MuKEn. These disruptive legal frameworks enable the Net-Zero goal to be achieved as early as 2040.

Efficiency performance requirements are heavily tightened whilst introducing new CO_2 performance metrics, along with an outright retrofit obligation for specific building types over 100kWh/m^2. This is coupled with an increase in renewable heat production requirements and the electricity self-production metric for retrofits. In order to fully synchronize standards between new and existing buildings, the EnDK move towards removing original MuKEn derating factors (initially ranging between 125-150%).

The CO_2 tax is heavily increased in the short-term, but will be relaxed in the long-term, as CO_2 emission targets are achieved ahead of schedule. The available budget and its reallocation of the continued Building Program behave similarly to NZ-50. A supplementary incentive program to increase the retrofit rate per depth (e.g. skipping at least two GEAK categories) and high incentives for self-consumption are introduced immediately. Cantonal zoning laws shift towards increased parcel utilization factors (density) for green buildings similar to NZ-50, but with extensive measures. Thus, the retrofit rate is increased, and voluntary building labels are in high demand.

The political tension, relating to the environment and economy, intensifies due to increasing neoliberal agendas in urban centers. To promote sustainable retrofitting, most urban municipalities are focused on relaxing rent controls coupled with loosening tenant laws relating to increased component pass-on rates and significantly reduced eviction notices. Due to increased rental prices based on real estate rental market liberalization, municipalities are forced to further step-in to subsidize 'green' affordable housing mandates for low-income renters. The increased retrofit activities are still mainly observed in urban centers, but retrofitting in rural areas is further promoted by incentive programs as it is difficult to demonstrate significant retrofitting value potential.

Expert elicitation workshop and interviews

The expert elicitation workshop successfully helped to validate the scenarios. The workshop promoted valuable discussions between experts with different competencies and responsibilities. From the discussions and feedback on the worksheets in the workshop, it was concluded that the scenario narratives are verified to be distinct from each other. The policy instrument toolkit is confirmed to be comprehensive, whereby individual instruments could be split into additional subgroups (e.g. specific cantonal tenancy regulations) for accuracy but can be generalized at the national level.

The expert opinions are relatively diversified for most proposed scenario-specific policy instrument trends. This disparity is very pronounced in the categories of real estate elements and financial incentives and building certificates. Many disagreements are expressed on the building certificate developments, as the majority expects them to increase in future.

Further, three interviews were conducted to obtain an overview of the current retrofitting landscape and the cantonal collaboration with the various investors. The main interview findings are presented in the following.

The cantonal authorities emphasize the implementation of SES-2050. Thus, they offer their financial support for consultations (e.g. GEAK Plus), or create information campaigns and organize public presentations. The cantonal authorities' focus on sustainable retrofitting is primarily directed towards financial incentives. Based on the HFM 2015, subsidies are mostly granted for individual components. However, the cantons do not have enormous budgets that allow them to turn previously unprofitable retrofitting projects into economically viable projects. In one of the two cantons, subsidies are said to be sufficient to provide financial security in approximately 50% of the cases.

The experts observed that the investor's mindset already shifted away from "one-to-one" heating substitutes to voluntary sustainable investments. Renewable technologies are the accepted standard for retrofitting, even if not required by law. In many cases, the entire system, instead of only meeting the legally required 10%, is designed to be powered by renewables. Often, this decision is based on the system costs incurred. Thus, it can be stated that the current legislation implies an indirect ban on fossil fuels. Further, for many investors, simply complying with the regulations is not innovative enough to meet future requirements.

The current retrofitting-related regulations are primarily seen as very diversified. It is required to harmonize or even combine instruments such as MuKEn or GEAK into a single policy instrument in the future. The introduction of the CO_2 tax led to changes towards a more sustainable sector and thus, the upcoming revision is expected to introduce stricter regulations. The consequences of the current tenancy law on the retrofitting behavior of investors are well known. Laws such as the LDTR in Geneva are particularly critical in driving the retrofitting behavior of real estate owners. However, this situation will not change in the

foreseeable future, given the political circumstances in Switzerland. The cantons' power is rather limited with regard to law amendments. Moreover, a large part of the population is politically in favor of affordable housing. Consequently, new approaches must be found to do justice to the different political interests.

Policy instrument evolutions and model implementation

The policy instruments implementation in the model are split between the asset-level (context parameters along with regulations, fiscal, and financial policies) and the portfolio-level (performance-based policies). Below we describe the evolutions of each policy instrument within each policy scenario.

While developing the scenarios, there were several policy instruments in which there were limitations for implementation in the model framework due to tractability issues. These are: zoning law densification bonus, rent controls / rent freezes, incentives per retrofitting depth, and portfolio reinvestment requirements.

- **Context parameters**

The results for the energy price and grid decarbonization developments are based on a comprehensive study published by in the context of the SES-2050[46]. The annual percentage changes in price are extracted from the year-to-year projections for heating oil, natural gas, and electricity. The starting levels are adapted to the 2019 levels, sourced from the Schweizerische Gesamtenergiestatistik 2019[47] (Swiss total energy statistics). The developed policy scenarios are aligned to the SES scenarios' parameters on energy carrier prices and grid decarbonization as such: BAU and NZ-50 Political measures (Politische Massnahmen POM) and NZ-40 New energy politics (Neue Energiepolitik NEP). In the SES-2050, the Business as Usual (Weiter wie bisher WWB) has similar energy carrier costs as to POM. The Feed-in-Tariff (export price) for electricity is assumed to be 1/3 of the electricity price in the scenario.

- **Regulations**
  - **Building energy code**
    - **Thermal energy efficiency (kWh/m^2):** Standards for building heating demands are predominantly for new buildings per type, with those for existing buildings derated to account for building construction complications (e.g. thermal bridges). We utilize the current (2020) regulation for new buildings in Switzerland and derate each building type with more stringency towards Net-Zero[16].
      - Derating factors:
        1. BAU: average value 36 kWh/m^2 (range 24-38 kWh/m^2 per building type)

- NZ-50: average value 30 kWh/m^2 (range 20-31 kWh/m^2 per building type)
- NZ-40: average value 24 kWh/m^2 (range 16-25 kWh/m^2 per building type)

- **CO_2 performance requirement (kgCO_2/m^2):** Emissions performance requirements for building heating demands are taken from the Swiss EPC (GEAK)[48] and SIA[49] for future target values for existing buildings considering both operational and embodied emissions aspects.
    - BAU: No limit
    - NZ-50: 20 kgCO_2/m^2
    - NZ-40: 10 kgCO_2/m^2
- **Fossil-fuel technology bans:** Bans on fuel oil and natural gas boilers are implied from BEC current standards and future outlooks[16].
    - BAU: No bans
    - NZ-50: Fuel oil ban in 2020
    - NZ-40: Fuel oil and natural gas ban in 2020.
- **Renewable heat requirements (%):** Annual non-fossil fuel heat production after a boiler is replaced, as defined in BEC regulations, are implied from BEC current standards and future outlooks[16]. Typically only refers to biomass boilers, heat pumps, and district-heating.
    - BAU: 10%
    - NZ-50: 40%
    - NZ-40: 80%
- **On-site electricity production requirements (W/m^2):** Annual renewable energy production requirements, as defined in BEC regulations, are implied from building energy code current standards and future outlooks[16]. Typically only refers to solar PV.
    - BAU: No requirement
    - NZ-50: 5 W/m^2
    - NZ-40: 10 W/m^2
- **Retrofit obligations (> kWh/m^2):** Existing building retrofit obligations currently do not exist but are assumed from BEC regulations future outlooks[16]. For NZ-40, any building that has a heating demand greater than 100 kWh/m^2 are requirement to achieve a lower value at the least cost.
    - Not implemented in model due to tractability issues

- **Real estate**
    - **Portfolio reinvestment requirements (%):** Minimum real estate portfolio reinvestment requirements are mandated by the national financial supervisory authority (FINMA) for all listed real estate funds[50]. Of the current 2020 level (2%), we project that they will have to increase to sufficiently allow for sufficient retrofitting activity.
        - Not implemented in model due to tractability issues

- **Component pass-on rates (%):** As the main instrument to regulate legally-allowed value-enhancing measures in existing buildings, component pass-on rates are largely regulated by legal precedents. They mandate the amount of component CAPEX that can be amortized to tenants in the rent. As such moving from the 2020 levels for each component, we decrease component pass on rates in NZ-50 (tenant-friendly) and increase them in NZ-40 (owner-friendly) based on recent reports[35,36].
    - BAU: 2020 levels
    - NZ-50: -10% for all components
    - NZ-40: +10% for all components
- **Rent controls (years):** One of the main rent control instruments is to freeze rents for a certain number of years post-retrofit. Based on recent case studies[51], we add a post-retrofit rent freeze of 5 years for NZ-50 (tenant-friendly).
    - Not implemented in model due to tractability issues

- **Financial incentives and fiscal instruments**
    - **CO_2 tax (CHF/tonCO_2):** CO_2 taxes on fossil-fuels for heating are a heavily politicized and active topic[52].
        - BAU: 120 CHF/tonCO_2 (constant at 2020 value)
        - NZ-50: 120 CHF/tonCO_2 (linear increase with overall 40%)
        - NZ-40: 145 CHF/tonCO_2 (linear increase with overall -33%)
    - **Incentives per retrofitting depth – linear and fixed (CHF / (% energy change)*m^2):** These can apply to achieving a certain retrofitting depth on a kWh/m^2 or kgCO_2/m^2 basis[53].
        - Not implemented in model due to tractability issues
    - **Incentives per component – linear and fixed (CHF/m^2 or CHF/kW or CHF/installation):** We utilize current standards and future outlooks from the cantonal incentive program[53]. These can apply to any retrofitting, conversion, or storage technology presented in Supplementary Table 2.
        - BAU: 2020 levels
        - NZ-50: 50% increase
        - NZ-40: 100% increase
    - **Solar photovoltaic incentives – linear and fixed (CHF/kW or CHF/installation):** We utilize current standards and future outlooks from the photovoltaic incentive program[54]. For simplicity, we assume a constant value for all sizes. Presented in Supplementary Table 2.
        - BAU: 2020 levels
        - NZ-50: 50% increase
        - NZ-40: 100% increase

Supplementary Table 1: Correlation matrix of policy instruments. Related to STAR Methods.

				BAU	NZ-50	NZ-40	Regulations																						Financial incentives and fiscal instruments											
							Building energy code													Real estate									CO_2 tax			Buildings program						Other		
							Thermal energy efficiency (kWh/m^2)			CO_2 performance ($kgCO_2/m^2$)		Technology bans / mandates		Renewable heat production requirements (%)		On-site electricity production requirements (%)		Retrofit obligations		Zoning law densification boni		Portfolio reinvesment (%)		Component pass-on rates (%)		Rent controls			CO_2 tax (EUR / $tonCO_2$)			Incentives per depth (fixed or linear)			Incentives per component (fixed or linear)			Feed-in-tariff for solar PV (EUR / kWh)		
			future level	Business-as-usual	Net-Zero 2050	Net-Zero 2040	a	b	c	a	b	a	b	a	b	a	b	a	b	a	b	a	b	a	b	a	b	c	a	b	c	a	b	c	a	b	c	a	b	c
Regulations	Building energy code	Thermal energy efficiency (kWh/m^2)	a: current level	■																																			
			b: slightly increased		■																																		
			c: strongly increased			■																																	
		CO_2 performance ($kgCO_2/m^2$)	a: no CO2 requirement	■			1	1	0																														
			b: CO2 requirement		■	■	0	1	2																														
		Technology bans / mandates	a: no bans	■			1	1	0	1	0																										
			b: ban for fossils (timing)		■	■	1	1	2	1	2																										
		Renewable heat production requirements (%)	a: current level	■			1	1	0	1	-1	1	0																								
			b: increased		■	■	0	1	2	-1	2	0	2																								
		On-site electricity production requirements (%)	a: current level	■			1	0	0	1	1	1	1	1	1																						
			b: increased		■	■	0	1	1	1	2	1	2	1	2																						
		Retrofit obligations	a: no obligations	■	■		1	1	1	1	1	1	1	1	1	1	1																				
			b: retrofit oblications			■	1	1	2	1	2	1	2	1	2	1	2																				
	Real estate	Zoning law densification boni	a: no boni	■			1	1	1	1	0	1	1	1	1	1	1	1	1																		
			b: densification boni		■	■	1	1	2	0	2	1	2	1	2	1	2	1	2																		
		Portfolio reinvesment (%)	a: current level	■	■		1	1	1	1	1	1	1	1	1	1	1	1	1	1	1																
			b: increased reinvestment			■	1	1	2	1	2	1	2	1	2	1	2	1	2	1	2																
		Component pass-on rates (%)	a: current level	■			1	0	-1	1	-1	1	-1	1	-1	1	-1	1	-1	1	0	1	1														
			b: increased (diff. degs.)		■	■	-1	1	2	-1	2	-1	2	-1	2	-1	2	0	2	0	2	1	2														
		Rent controls	a: current controls	■			1	1	1	1	1	1	1	1	1	1	1	1	-1	1	0	1	0	1	-1												
			b: stronger controls				1	0	-1	1	-1	1	-1	1	-1	1	-1	0	-1	0	1	0	0	-1	-1												
			c: weaker controls (to diff. degs.)		■	■	-1	0	1	-1	1	-1	1	-1	1	-1	1	0	1	0	1	0	0	-1	2												
Financial incentives and fiscal instruments	CO_2 tax	CO_2 tax (EUR / $tonCO_2$)	a: current level	■			1	1	-1	1	-1	1	-1	1	-1	1	-1	1	-1	1	-1	1	0	1	-1	1	0	0									
			b: steady increased		■		0	1	1	1	1	1	1	1	1	0	1	0	1	0	1	1	1	1	1	1	1	0									
			c: up & down			■	-1	1	2	-1	2	-1	2	-1	2	-1	2	-1	2	-1	2	0	1	-1	2	0	1	2									
	Buildings program	Incentives per depth (fixed or linear)	a: current level		■		1	1	-1	1	0	1	0	1	-1	1	0	1	1	1	0	1	0	1	0	1	0	0	1	1	1						
			b: increased			■	0	1	2	0	2	0	2	1	1	1	2	1	2	0	1	0	1	0	1	0	-1	0	1	1	1						
			c: decreased	■			1	-1	-2	0	-1	1	-1	1	-1	1	-1	1	-1	1	-1	1	-1	1	-1	0	1	-1	0	1	-1						
		Incentives per component (fixed or linear)	a: current level		■		1	0	-1	1	0	1	0	1	0	1	0	1	0	1	0	1	0	1	0	1	0	0	1	1	0	1	0	0			
			b: increased			■	0	1	2	0	2	0	2	0	2	0	2	0	2	0	2	0	1	0	1	0	-1	1	1	2	2	0	2	-2			
			c: decreased	■			1	-1	-2	1	-2	0	-1	0	-1	0	-2	0	-2	0	1	0	-1	0	-1	0	1	-1	1	0	1	0	-2	2			
	Other	Feed-in-tariff for solar PV (EUR / kWh)	a: current level		■		1	0	0	1	0	1	0	1	0	1	-1	1	0	1	0	1	0	1	0	1	0	0	1	0	0	1	0	0	1	0	0
			b: increased			■	0	0	1	0	1	0	1	0	1	0	2	0	1	0	-1	0	1	0	-1	0	1	-1	0	0	-1	0	1	1	0	1	-1
			c: decreased	■			0	0	-1	0	-1	0	-1	0	-1	0	-2	0	-1	0	1	0	-1	0	1	0	-1	1	0	0	1	0	1	1	0	-1	2

correlation matrix legend:

score	thematic consistency
0	**no relation**
1	**consistent**
2	**consistent and reinforcing**
-1	**partly inconsistent**
-2	**absolutely inconsistent**

Correlation matrix presents the interactions between all policy instruments considered in the scenarios.

Supplementary Table 2: Technology linear cost and incentive values per policy scenario. Related to STAR Methods.

**Tech.**	**Linear cost 2021**	**Learning Rate (Gamma)**	**BAU inct. 2021**	**NZ-50 inct. 2021**	**NZ-40 inct. 2021**	**BAU inct. (%)**	**NZ-50 inct. (%)**	**NZ-40 inct. (%)**
**Conv.**	**(EUR/kW)**		**(EUR/kW)**	**(EUR/kW)**	**(EUR/kW)**			
GSHP	1,825	0.035	360	550	820	20%	30%	45%
ASHP	860	0.027	210	300	400	24%	35%	47%
Bio Boiler	825	0	200	300	400	24%	36%	48%
CHP	900	0.005	200	300	400	22%	33%	44%
PV	1600	0.021	394	591	788	25%	37%	49%
**Stor.**	**(EUR/kWh)**		**(EUR/kWh)**	**(EUR/kWh)**	**(EUR/kWh)**			
Battery	370	0.019	0	100	180	0%	27%	49%
**Ret.**	**(EUR/m^2)**		**(EUR/m^2)**	**(EUR/m^2)**	**(EUR/m^2)**			
Window	586-709	0	40	60	80	6-7%	8-11%	11-14%
Facade	150-240	0	40	60	80	17-27%	25-40%	33-53%
Roof	150-240	0	40	60	80	17-27%	25-40%	33-53%

Full detail on the input cost parameters is presented in the original MANGOret model formulation[55]. Cost evolutions for technologies with learning curves are presented in Extended Data Fig 4. All incentives are implemented as constant percentages to the linear cost of technologies, thus if applicable incentives follow the same paths as the learning curves. Retrofit technology costs are presented here per component as ranges for simplicity. The possible technological set varies between (in order of low to high cost), Windows: Plastic & Wood-Aluminium and Roof & Façade insulation: XPS, EPS, and Stonewool.

Supplementary results

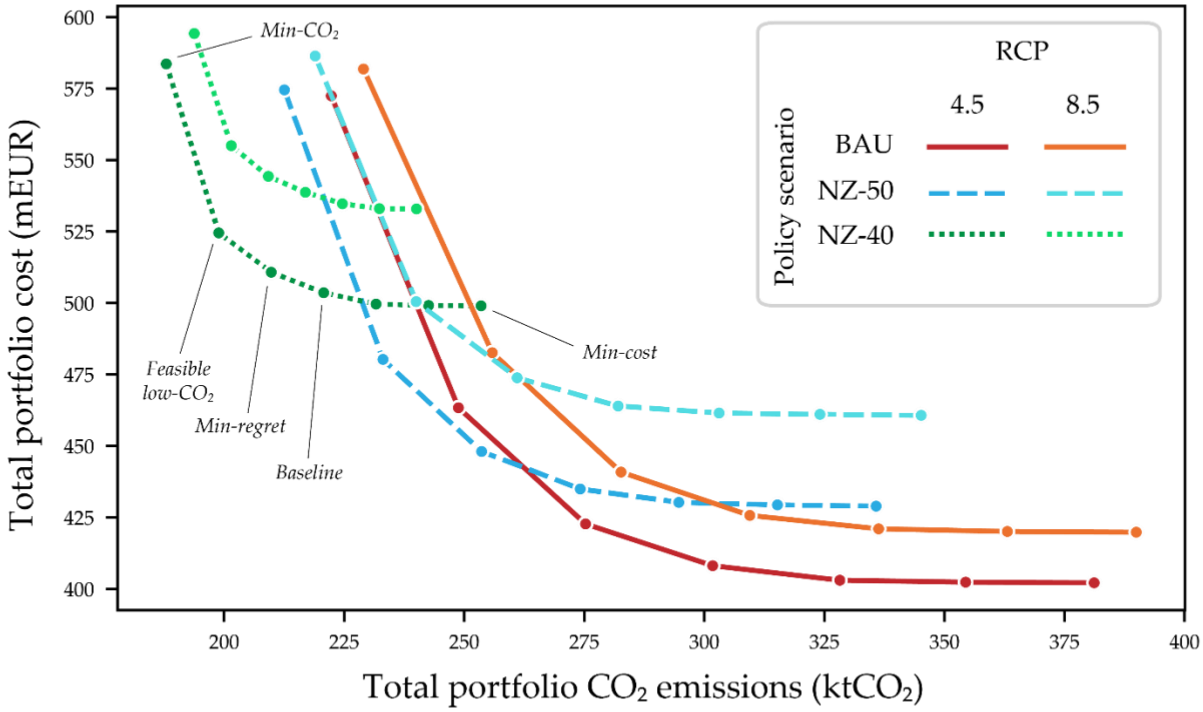

Supplementary Figure 1 | Optimal portfolio scenarios under policy conditions and RCPs. Pareto fronts for the total aggregated portfolio 30-year costs and CO_2 emission with RCPs 4.5 and 8.5. RCP 8.5 consistently increases costs for the same levels of emissions with regards to RCP 4.5. Related to STAR Methods.

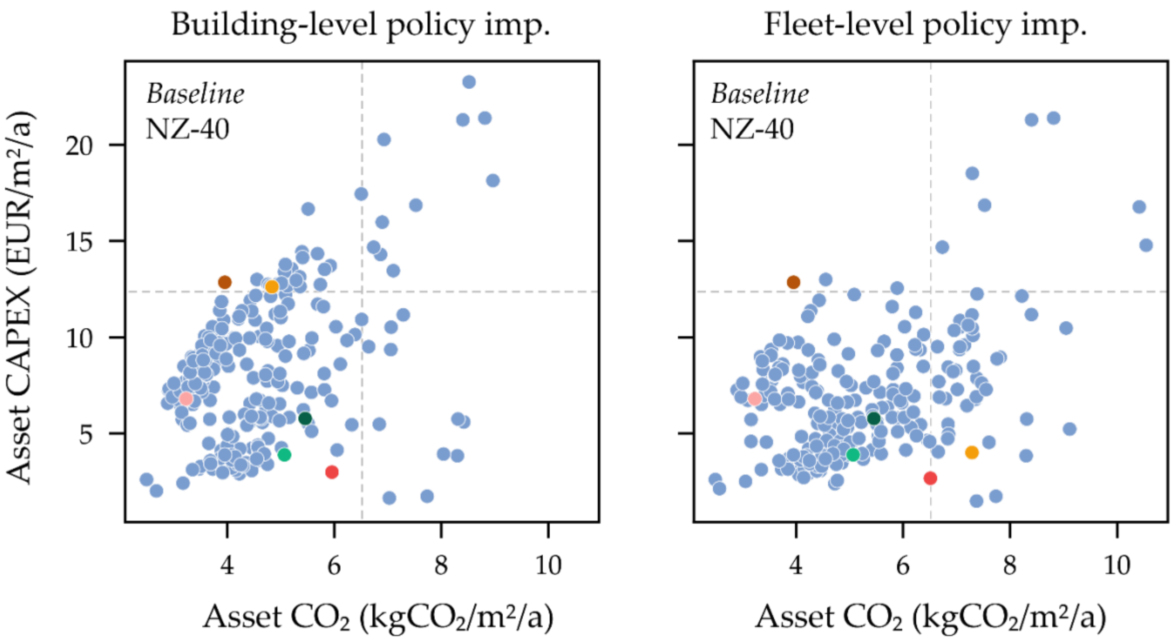

Supplementary Figure 2 | Asset-level performance in *Baseline* NZ-40 strategy across policy implementation approaches building- (left) vs. fleet-level (right). Asset's average annual CAPEX investment and CO_2 emissions are shown. Six assets are highlighted in color to illustrate their movement between the strategies, categorized as: "hard-to-decarbonize": brown and yellow, "low-hanging-fruit": dark and light green, "no change": red and pink. Related to STAR Methods.

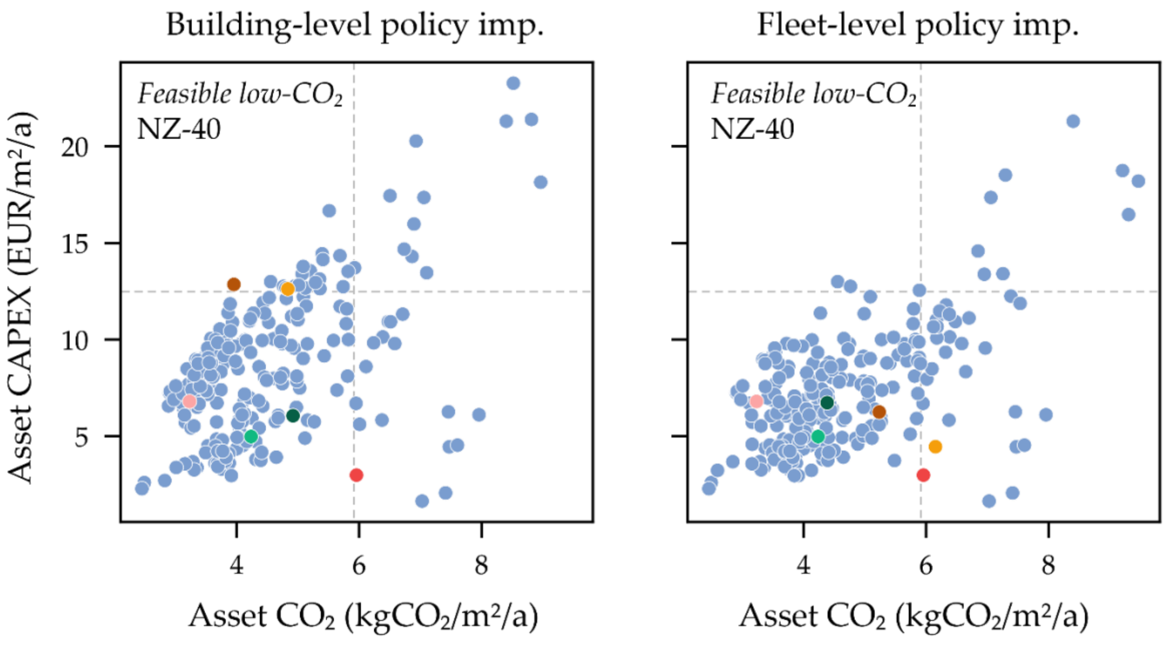

Supplementary Figure 3 | Asset-level performance in *Baseline* NZ-40 strategy across policy implementation approaches building- (left) vs. fleet-level (right). Asset's average annual CAPEX investment and CO_2 emissions are shown. Six assets are highlighted in color to illustrate their movement between the strategies, categorized as: "hard-to-decarbonize": brown and yellow, "low-hanging-fruit": dark and light green, "no change": red and pink. Related to STAR Methods.

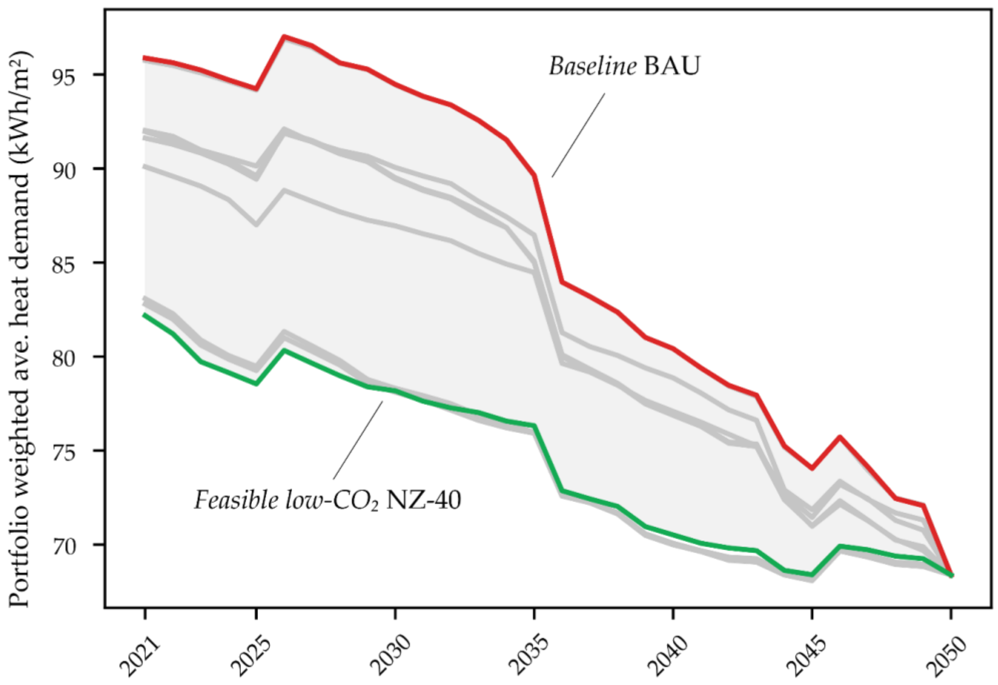

Supplementary Figure 4 | Heating energy demand reduction pathways. Aggregated portfolio weighted average results compared between the *Baseline* BAU (red) and *Feasible low-CO_2* NZ-40 (green) scenarios (RCP 4.5, fleet-level policy implementation). All other scenarios lie in the gray zone between. Related to STAR Methods.

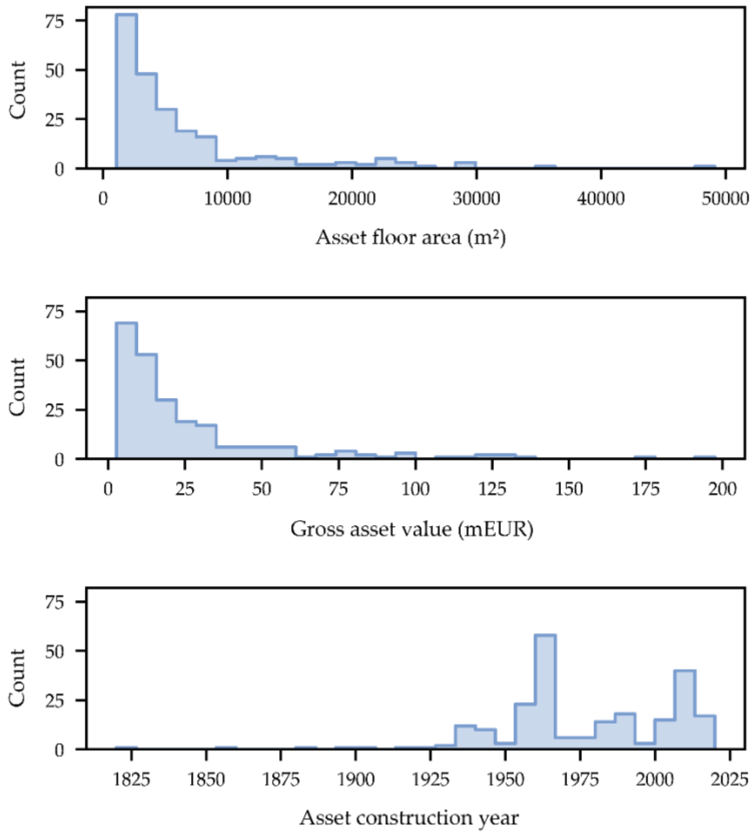

Supplementary Figure 5 | Histograms with basic information on aggregated portfolio. Asset floor area, gross asset value, and construction years are presented. Related to STAR Methods.

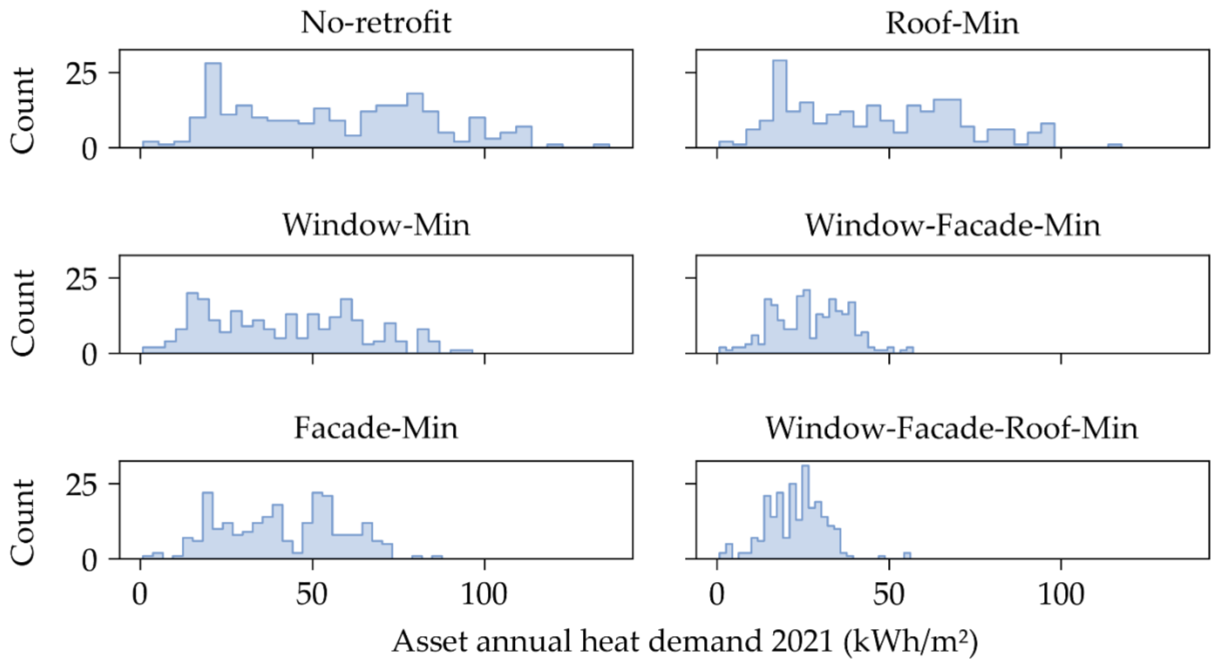

Supplementary Figure 6 | Heating energy demand histograms of asset renovation scenarios in first year of analysis (2021). Minimum retrofitting depth shown (not Target) for RCP 4.5. The variance of retrofit heating demand reductions for unique buildings is shown. Related to STAR Methods.

References

1. van Notten, P. (2006). Scenario development: a typology of approaches. In Think Scenarios, Rethink Education (OECD Publishing), pp. 66–92. https://doi.org/10.1787/9789264023642-6-en.

2. IPCC (2000). Special Report on Emissions Scenarios.

3. SFOE (2018). Energy Strategy 2050: Once the New Energy Act Is in Force.

4. European Commission (2020). A Renovation Wave for Europe - greening our buildings, creating jobs, improving lives.

5. European Commission (2020). A Renovation Wave initiative for public and private buildings.

6. Gatt, D., Yousif, C., Cellura, M., Camilleri, L., and Guarino, F. (2020). Assessment of building energy modelling studies to meet the requirements of the new Energy Performance of Buildings Directive. Renewable and Sustainable Energy Reviews *127*, 109886. 10.1016/j.rser.2020.109886.

7. Robiou Du Pont, Y., Jeffery, M.L., Gütschow, J., Rogelj, J., Christoff, P., and Meinshausen, M. (2017). Equitable mitigation to achieve the Paris Agreement goals. Nat Clim Chang *7*, 38–43. 10.1038/nclimate3186.

8. Intergovernmental Panel on Climate Change (IPCC) (1992). Climate Change: The 1990 and 1992 IPCC Assessments.

9. United Nations (UN) (1998). Kyoto Protocol to the United Nations Framework Convention on Climate Change 10.1093/jel/10.1.215.

10. Mahmoud, M., Liu, Y., Hartmann, H., Stewart, S., Wagener, T., Semmens, D., Stewart, R., Gupta, H., Dominguez, D., Dominguez, F., et al. (2009). A formal framework for scenario development in support of environmental decision-making. Environmental Modelling and Software *24*, 798–808. 10.1016/j.envsoft.2008.11.010.

11. Swart, R.J., Raskin, P., and Robinson, J. (2004). The problem of the future: Sustainability science and scenario analysis. Global Environmental Change *14*, 137–146. 10.1016/j.gloenvcha.2003.10.002.

12. Kishita, Y., Hara, K., Uwasu, M., and Umeda, Y. (2016). Research needs and challenges faced in supporting scenario design in sustainability science: a literature review. Sustain Sci *11*, 331–347. 10.1007/s11625-015-0340-6.

13. Robinson, J.B. (1990). Futures under glass: A recipe for people who hate to predict. Futures *22*, 820–842. 10.1016/0016-3287(90)90018-D.

14. Romano, E., Cozza, S., Chambers, J., Schneider, S., Yilmaz, S., and Patel, M. (2019). Definition of reference scenario to be used by all FEEB&D partners.

15. Woodward, R.T., and Bishop, R.C. (1997). How to Decide When Experts Disagree: Uncertainty-Based Choice Rules in Environmental Policy. Land Econ *73*, 492–507. 10.2307/3147241.

16. EnDK (2015). Model regulations of the cantons in the energy sector 2014 (Mustervorschriften der Kantone im Energiebereich (MuKEn) 2014).

17. Prognos AG (2012). Die Energieperspektiven für die Schweiz bis 2050.

18. Schweizerischer Bundesrat (2017). Botschaft zur Totalrevision des CO2-Gesetzes nach 2020.

19. Bundesamt für Umwelt (BAFU) (2020). Emissionen von Treibhausgasen nach revidiertem CO2 Gesetz und Kyoto-Protokoll, 2. Verpflichtungsperiode (2013-2020).

20. Schweizerischer Bundesrat (2014). Bundesgesetz über die Reduktion der CO2 - Emissionen (CO2-Gesetz).

21. Bundesamt für Umwelt (BAFU) Erhebung der CO2-Abgabe auf Brennstoffen. https://www.bafu.admin.ch/bafu/de/home/themen/klima/fachinformationen/klimapolitik/co2-abgabe/erhebung-der-co2-abgabe-auf-brennstoffen.html.

22. Schweizerischer Bundesrat (2016). Energiegesetz (EnG).

23. Schwarz, M., Nakhle, C., and Knoeri, C. (2020). Innovative designs of building energy codes for building decarbonization and their implementation challenges. J Clean Prod *248*, 119260. https://doi.org/10.1016/j.jclepro.2019.119260.

24. Schweizerischer Ingenieur- und Architektenverein (SIA) (2016). SIA 380/1.

25. MuKEn AG (2016). Herleitung / Übersicht und Grenzwertentwicklung von der SIA 380/1:(2009) auf die neue Norm SIA der SIA 380/1:(2016).

26. Wüest Partner AG (2020). Auswirkungen der Heizungsersatzregelung der MuKEn auf die Wahl des Heizträgers.

27. Bauen, I. (2018). Nationale und internationale Labels für nachhaltiges Bauen. 36–41.

28. European Parliament (2018). Directive (EU) 2018/844.

29. Schmid, N., Haelg, L., Sewerin, S., Schmidt, T.S., and Simmen, I. (2020). Governing complex societal problems: The impact of private on public regulation through technological change. Regul Gov. https://doi:10.1111/rego.12314.

30. Bundesamt für Umwelt (BAFU) (2018). Switzerland's climate policy - Implementation of the Paris Agreement.

31. Bundesamt für Energie (BFE) (2012). Energiestrategie 2050: Erstes Massnahmenpaket.

32. UNEP (2021). 2021 Global Status Report for Buildings and Construction: Towards a Zero-emission, Efficient and Resilient Buildings and Construction Sector.

33. Schweizerischer Bundesrat (2020). Verordnung über die Miete und Pacht von Wohn- und Geschäftsräumen (VMWG).

34. Mieterinnen- und Mieterverband Ostschweiz (2018). Umbauten, Renovationen, Totalsanierungen.

35. King, M., Heim, T., and Menti, U. (2019). Value-enhancing and value-maintaining investments in comprehensive refurbishments (Werterhaltende und wertvermehrende Investitionen bei umfassenden Sanierungen).

36. Tenant law practice (Mietrechtpraxis) (2020). Examples of frequent replacement investments (Beispiele häufiger Ersatzinvestitionen).

37. Mieterinnen- und Mieterverband Anfangsmietzins. https://www.mieterverband.ch/mv/mietrecht-beratung/ratgeber-mietrecht/top-themen/anfangsmietzins.html.

38. Bundesamt für Wohnungswesen (BWO) Erhalt von Wohnraum. https://www.bwo.admin.ch/bwo/de/home/Wohnungsmarkt/buchungsplattformen/themen/erhalt_von_wohnraum.html#par_headline.

39. Schweizerischer Bundesrat (2020). Bundesverfassung der Schweizerischen Eidgenossenschaft (BV).

40. Kernen, B. (2011). Das Gesetz über die Abbrüche, die Umbauten und Renovationen von Wohnhäusern (LDTR) im Kanton Genf und seine Auswirkungen auf den Immobilienmarkt.

41. Schweizerischer Bundesrat (2017). Obligationenrecht (OR).

42. Mieterinnen- und Mieterverband Deutschschweiz Kündigung durch die Vermieterschaft: Anfechtung durch Mieter*innen.

43. Schweizerischer Bundesrat (2008). Bundesratsbeschluss über die Allgemeinverbindlicherklärung des Rahmenmietvertrages für die Westschweiz und über die Abweichung von zwingenden Bestimmungen des Mietrechts.

44. Schweizerischer Bundesrat Allgemeinverbindlichkeit des Rahmenmietvertrags für die Westschweiz erneuert. https://www.admin.ch/gov/de/start/dokumentation/medienmitteilungen.msg-id-53423.html.

45. Robinson, J.B. (1982). Energy backcasting A proposed method of policy analysis. Energy Policy *10*, 337–344. 10.1016/0301-4215(82)90048-9.

46. econcept AG (2017). Kosten und weitere Auswirkungen energiepolitischer Szenarien.

47. Bundesamt für Energie (BFE) (2019). Schweizerische Gesamtenergiestatistik 2019.

48. EnDK (2021). Standardization of the GEAK (The building energy certificate of the canton) (Normierung des GEAK (Der Gebäudeenergieausweis der Kantone)).

49. SIA (2000). SIA 381/1: Building materials and products - Thermal and moisture performance - Tabulated design values (Baustoffe und -produkte - Wärme- und feuchteschutztechnische Eigenschaften - Tabellierte Bemessungswerte).

50. SSF (2019). EU Action Plan on Sustainable Finance: Effects on Swiss financial institutions.

51. Government Council of the Canton of Basel-City (Regierungsrat des Kantons Basel-Stadt) (2020). Cantonal popular initiative "Yes to REAL housing protection" Report on legal admissibility and further procedure (Kantonale Volksinitiative «Ja zum ECHTEN Wohnschutz» Bericht über die rechtliche Zulässigkeit und zum weiteren Verfahren).

52. EnDK, and SFOE (2020). State of Energy and Climate Policy in the Cantons 2020 (Stand der Energie- und Klimapolitik in den Kantonen 2020).

53. SFOE, and EnFK (2016). Harmonized funding model of the cantons (Harmonisiertes Fördermodell der Kantone (HFM 2015)).

54. Pronovo (2021). Directive on the Energy Subsidies Ordinance - Photovoltaics (Richtlinie zur Energieförderungsverordnung (EnFV) - Photovoltaik).

55. Petkov, I., Mavromatidis, G., Knoeri, C., Allan, J., and Hoffmann, V.H. (2022). MANGOret: An optimization framework for the long-term investment planning of building multi-energy system and envelope retrofits. Appl Energy *314*, 118901. 10.1016/J.APENERGY.2022.118901.
