# Supplementary material for: Decarbonizing real estate portfolios considering optimal retrofit investment and policy conditions to 2050
Source: iScience. 2023 Apr 8;26(5):106619. doi: 10.1016/j.isci.2023.106619 (PMC10165412; doi:10.1016/j.isci.2023.106619)
Supplement: Data S1. Extended data for making the figures in the main text [file mmc2.pdf]

Extended Data Figures

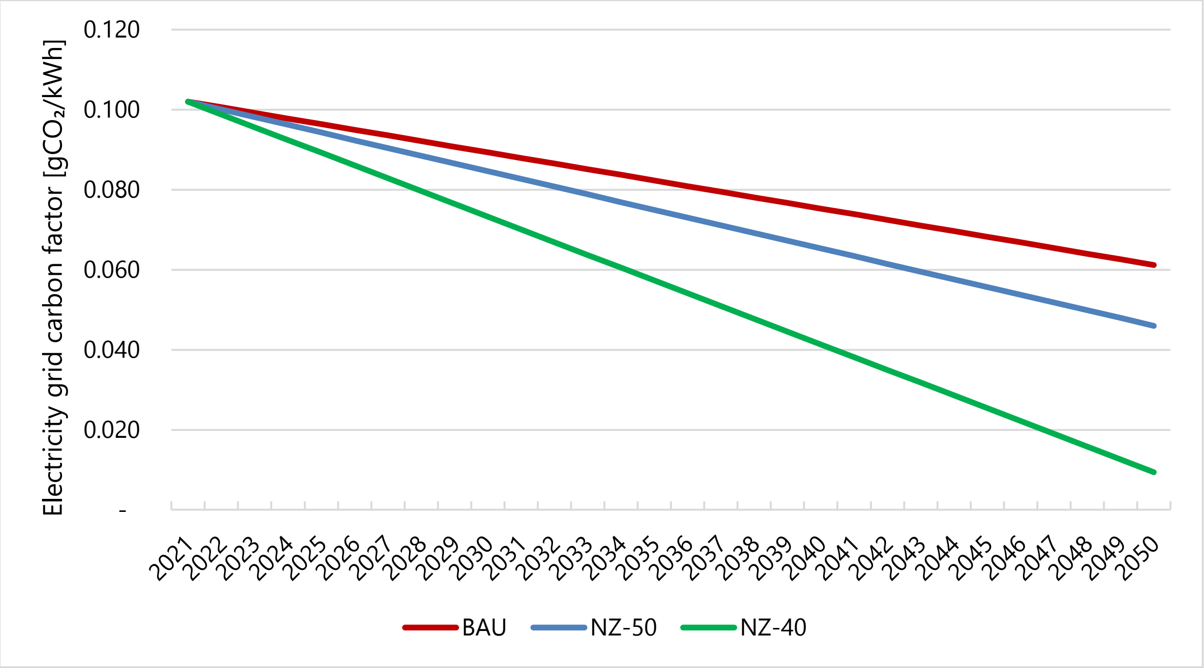

Extended Data Figure 1 | Electricity grid lifecycle CO_2 factors under policy scenarios (derived from Swiss Energy Strategy 2050). The national Swiss Energy Strategy 2050[11,90] (SES 2050) grid-decarbonization scenarios are linked to the policies developed here, with BAU associated to the SES 2050 POM scenario and NZ-40 associated to the SES 2050 POM scenario, with the NZ-50 as the "middle-path".

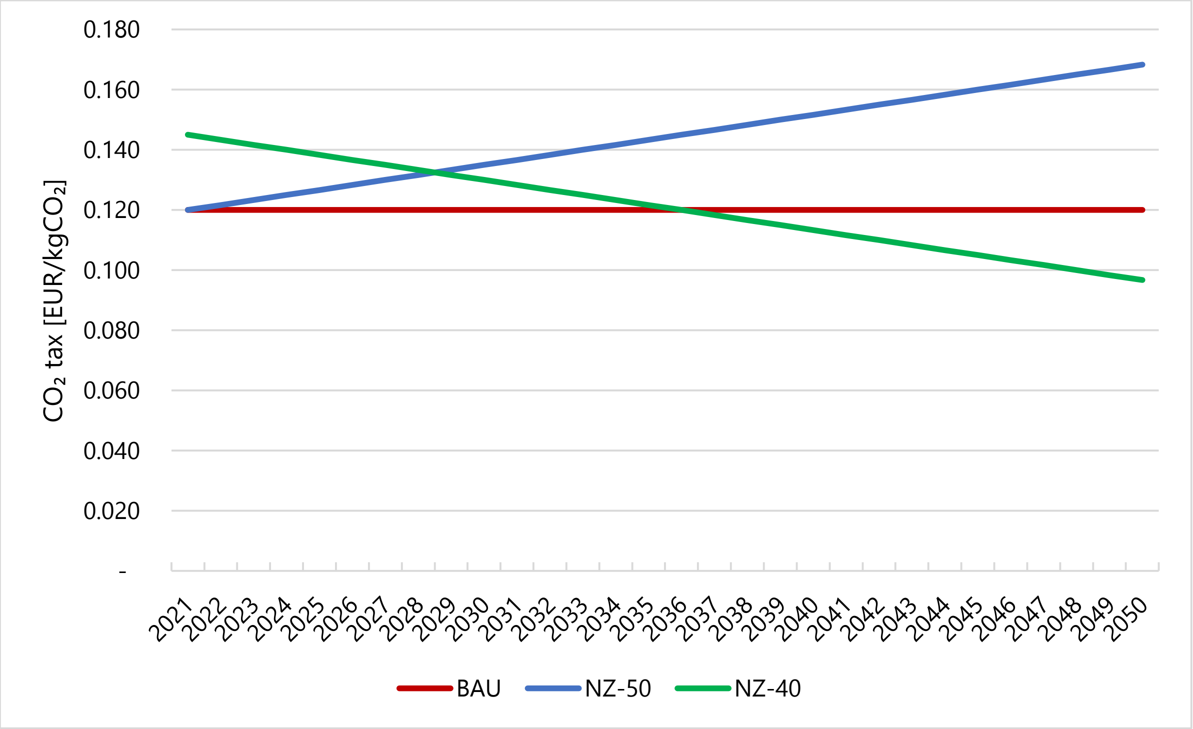

Extended Data Figure 2 | CO_2 tax developments under policy scenarios.

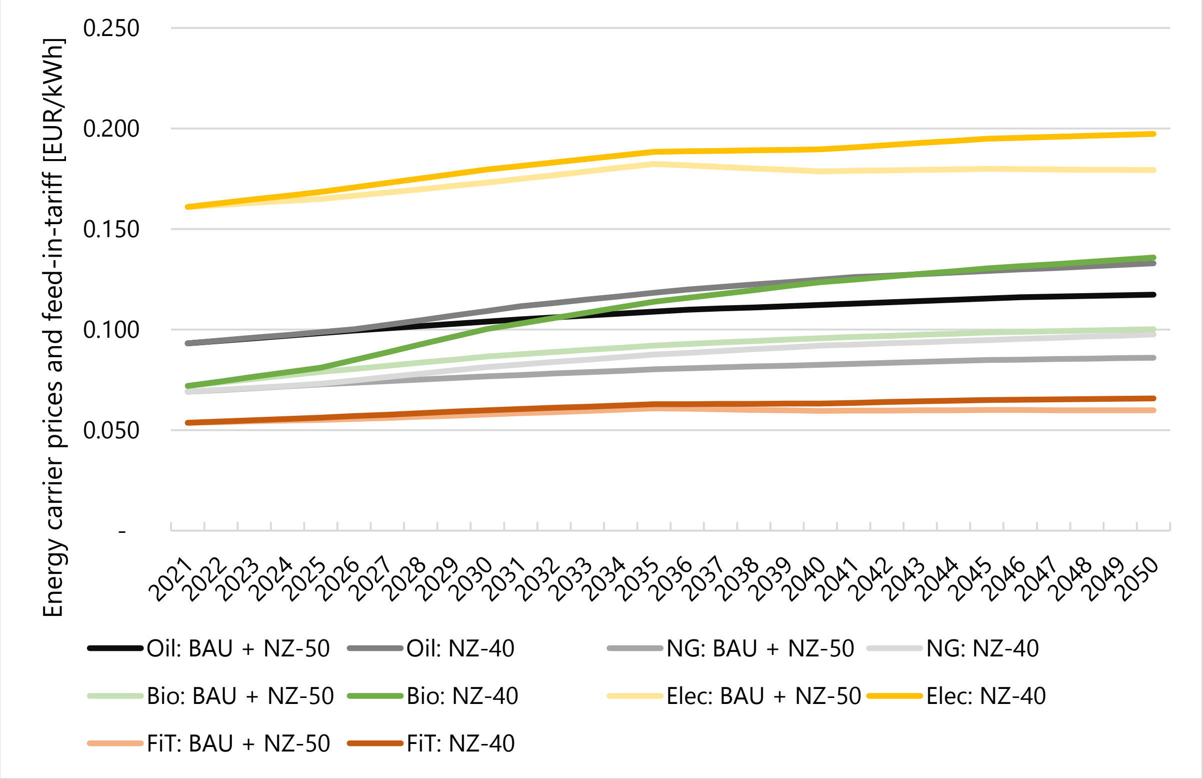

Extended Data Figure 3 | Energy carrier prices and feed-in-tariff under policy scenarios.

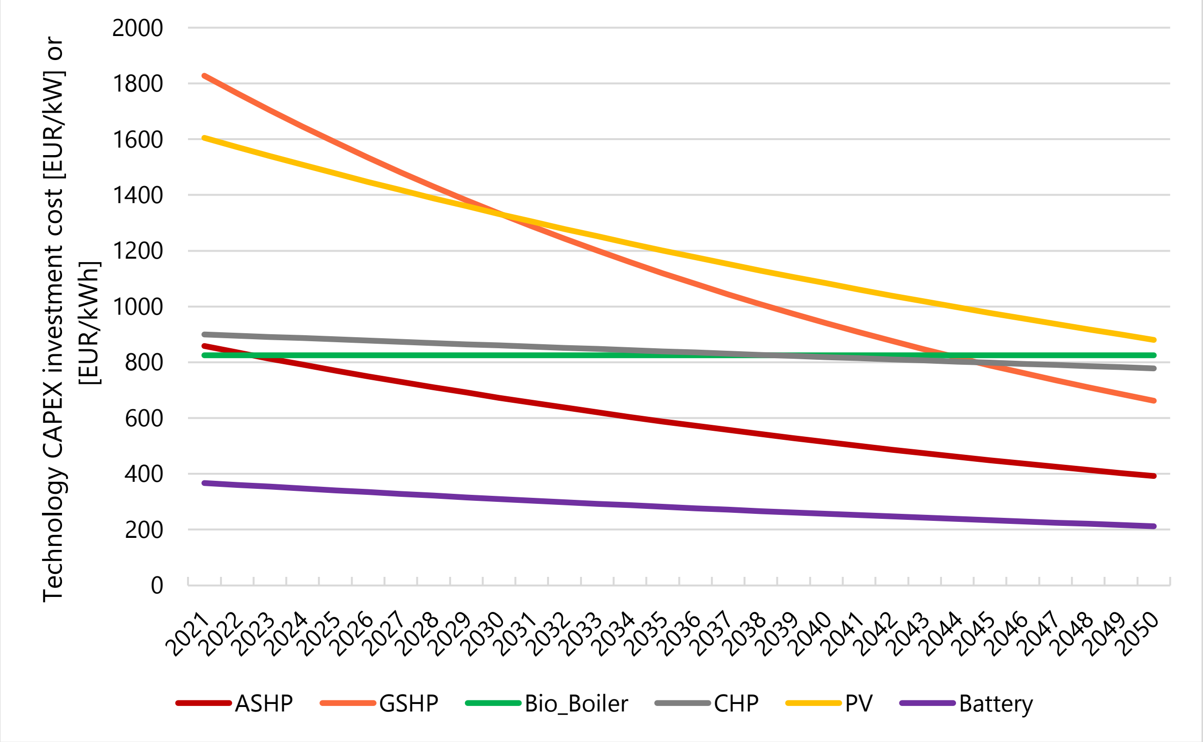

Extended Data Figure 4 | Selected technology CAPEX investment cost evolutions. Data is presented in Supplementary Table 2.

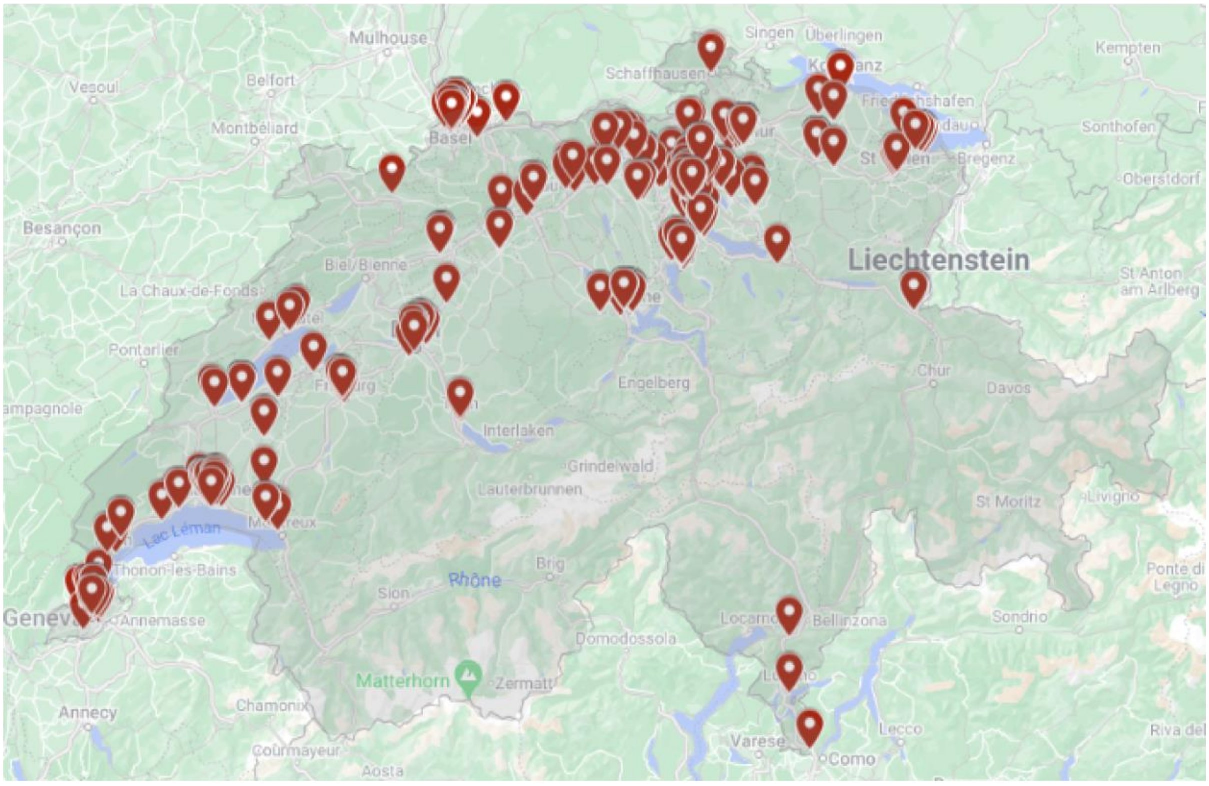

Extended Data Figure 5 | Asset geographic location in the Swiss portfolio case study.
